# Small molecule screening identifies cytotoxic endoplasmic reticulum-associated degradation inhibitors in multiple myeloma

**DOI:** 10.1038/s41419-026-08526-2

**Published:** 2026-03-09

**Authors:** Erin M. Kropp, Sho Matono, Olivia Y. Wang, Aaron M. Robida, Malathi Kandarpa, Jineigh L. Grant, Bryndon J. Oleson, Andrew Alt, Moshe Talpaz, Matthew J. Pianko, Qing Li

**Affiliations:** 1 Department of Internal Medicine, Division of Hematology/Oncology, University of Michigan, Ann Arbor, MI USA; 2 Veterans Affairs Ann Arbor Healthcare System, Medicine Service (111), Ann Arbor, MI USA; 3 Department of Cell and Developmental Biology, University of Michigan, Ann Arbor, MI USA; 4 Center for Chemical Genomics, Life Sciences Institute, University of Michigan, Ann Arbor, MI USA; 5 Department of Molecular, Cellular, and Developmental Biology, University of Michigan, Ann Arbor, MI USA; 6 Rogel Cancer Center, University of Michigan, Ann Arbor, MI USA

**Keywords:** High-throughput screening, Myeloma, Endoplasmic reticulum, Apoptosis, Stress signalling

## Abstract

Multiple myeloma (MM) is an incurable plasma cell neoplasm that is highly reliant on endoplasmic reticulum-associated degradation (ERAD) to maintain protein homeostasis. Disrupting ERAD has been proposed as a therapeutic strategy to overcome proteasome inhibitor resistance; however, the identification of novel inhibitors has been limited. To address this, we conducted a cell-based high-throughput screen using the FDA repurposing library and identified omaveloxolone (RTA408) as a potent ERAD inhibitor that selectively impairs the degradation of ER luminal and membrane substrates, without affecting the degradation of key cytosolic proteins that are implicated in disease relapse. Surprisingly, although ER stress response pathways are activated after ERAD inhibition in MM, we find that apoptosis is mediated by altered lipid raft organization, leading to aberrant activation of the death-inducing signaling complex (DISC) and caspase 8 in the extrinsic apoptotic pathway. Notably, ERAD inhibition by RTA408 is cytotoxic to primary malignant plasma cells, including those resistant to proteasome inhibitors, and demonstrates in vivo anti-myeloma activity. Our findings establish a novel ERAD inhibitor, which is a valuable tool to dissect ERAD biology, and provide pre-clinical evidence for RTA408 as a therapeutic agent in MM.

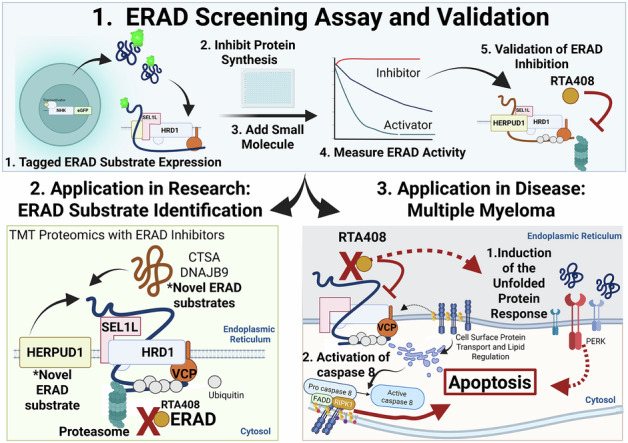

## Introduction

Multiple myeloma is the second most common hematologic malignancy and is characterized by high paraprotein secretion in over 95% of cases [1, 2]. Paraproteins are monoclonal immunoglobulins that are synthesized and modified in the endoplasmic reticulum (ER). Immunoglobulin synthesis is subject to inherent protein misfolding, which, if unabated, can lead to ER stress, activation of the unfolded protein response, and cell death [3, 4]. To manage this, malignant plasma cells are highly reliant on ER stress response pathways, including ER-associated degradation (ERAD). ERAD is a multiprotein complex that recognizes and degrades misfolded or target proteins in the ER [5]. ERAD recognizes protein targets in the ER and translocates them to the cytosol to be degraded by the ubiquitin-proteasome system (UPS). Proteasome inhibitors (PIs) disrupt the UPS, inhibiting ERAD substrate degradation, which results in both ER stress and pro-apoptotic signaling [3, 6]. While PIs have contributed to an improvement in MM overall survival from 2–3 years to 6.5–10 years, almost all patients develop PI resistance, resulting in relapsed or refractory (R/R) disease [7, 8]. Targeting alternative proteins in the ERAD complex has been proposed as a therapeutic strategy to overcome resistance [9, 10]. However, clinical development of such inhibitors has been hindered by off-target toxicities, poor pharmacodynamics, and lack of target specificity [9, 11–13]. Thus, the identification of novel small molecule modulators of ERAD activity that are safe, potent and specific to ER protein degradation is needed to develop therapeutic strategies for MM.

We developed a high-throughput cell-based small molecule drug screen to identify modulators of ERAD protein degradation and screened the FDA repurposing library. Here we report a novel compound, omaveloxolone (RTA408), which inhibits ERAD, induces the unfolded protein response, and leads to rapid induction of pro-apoptotic signaling in MM. RTA408 was developed to promote nuclear factor erythroid 2-related factor 2 (NRF2) activity by inhibiting NRF2 constitutive degradation by KEAP1-CUL3 E3 ligase and is approved for the treatment of Friedreich’s ataxia [14, 15]. However, we found that RTA408 is cytotoxic to MM cells independent of KEAP1, supporting a novel function as an ERAD inhibitor that is independent of NRF2 activation.

Using our ERAD inhibitor in combination with known modulators of ERAD activity, we were able to identify novel ERAD substates in MM and illustrate that differential targeting of ERAD components has distinct effects on ER and cytosolic protein homeostasis. Importantly, we find that differential targeting of ERAD by RTA408 depletes key cytosolic signaling proteins, such as C-MYC and NFkB, which have previously been implicated in treatment resistance or relapse and accumulate with PI inhibitors [16–18]. In addition, we unexpectedly uncovered the mechanism of cell death mediated by ERAD inhibition in MM, which was independent of the unfolded protein response, but instead requires activation of caspase 8. Although PI treatment has been shown to activate caspase 8 [6, 19–21], our studies show that ERAD inhibition is associated with altered lipid rafts and intrinsic activation of the death-induced signaling complex (DISC), which induces apoptosis in MM cells. These studies provide mechanistic insight into ERAD regulation in MM cells and pre-clinical evidence supporting ERAD inhibition as a therapeutic approach in R/R MM.

## Methods

### Cell lines

MM.1S, RPMI8226, MM.1 R, U266B1 were obtained from ATCC. KMS12-BM, AMO1, and INA-6 cells were obtained from DSMZ. NCIH929, OPM2, and KMS11 cells were a gift from the Talpaz lab. Cultures were routinely tested for mycoplasma contamination (Invivogen). MM.1S, RPMI8226, U226B1 were cultured in Roswell Park Memorial Institute (RPMI) 1640 growth media with 10% Fetal Bovine Serum (FBS; Fisher) and 1x Penicillin-Streptomycin-Glutamine (Gibco); KMS12-BM, AMO1 and INA-6 were cultured as above but with 20% FBS. INA-6 was maintained in 10 ng/mL recombinant human IL-6 (Peprotech). K562 cells expressing INSIG-GFP, RTA^E177Q^-GFP, and uGFP were a gift from the Baldridge lab. Proteasome-resistant AMO1 cell lines were developed by culturing with a gradual increase in BOR concentration as previously described [6, 22]. All cultured cells were maintained at 37°C with 5% CO_2_; cells were plated at 5e5 cells/mL unless specified.

#### Small molecule treatment

Selected lead small molecule inhibitors were reordered/obtained commercially as described in the key resource table. For commercially acquired compounds, small molecules were dissolved in DMSO with a final concentration ≤0.1% DMSO. Emetine was dissolved in tissue culture-grade H_2_O with a final concentration of ≤0.1%.

#### GFP-tagged ERAD substrate steady state degradation

Expression of NHK-GFP, INSIG-GFP, RTA^E177Q^-GFP, and uGFP in K562 cells was induced with 0.75 µg/mL doxycycline for 16 h. Doxycycline was removed, and K562 cells were plated with 20 µM emetine in phenol red-free RPMI 10% FBS and small molecule inhibitors at specified concentrations for select timepoints between 0-21 hours. DAPI (1 µg/mL) was added for dead cell exclusion and cells were immediately analyzed by flow cytometry (BD LSRFortessa).

### Xenograft experiments

NOD.CG-Prkdc^scid^Il2rg^tm1Wjl^/SzJ (NSG) mice were obtained from The Jackson Laboratory and maintained in-house. All animal studies were performed in accordance with IACUC guidelines and all mice were sacrificed at or prior to the development of symptomatic myeloma. Male mice between 6-10 weeks of age were utilized for RPMI8226 xenograft transplant studies as previously described [23, 24]. Mice received 175 cGy whole body irradiation (Kimtron Medical IC-320) followed by transplantation of 5e6 RPMI8226 cells with serial imaging and monitoring. All mice were maintained within the recommended tumor burden and survival endpoints per institutional regulations.

## Results

### Identification of ERAD inhibitors

**Fig. 1:**
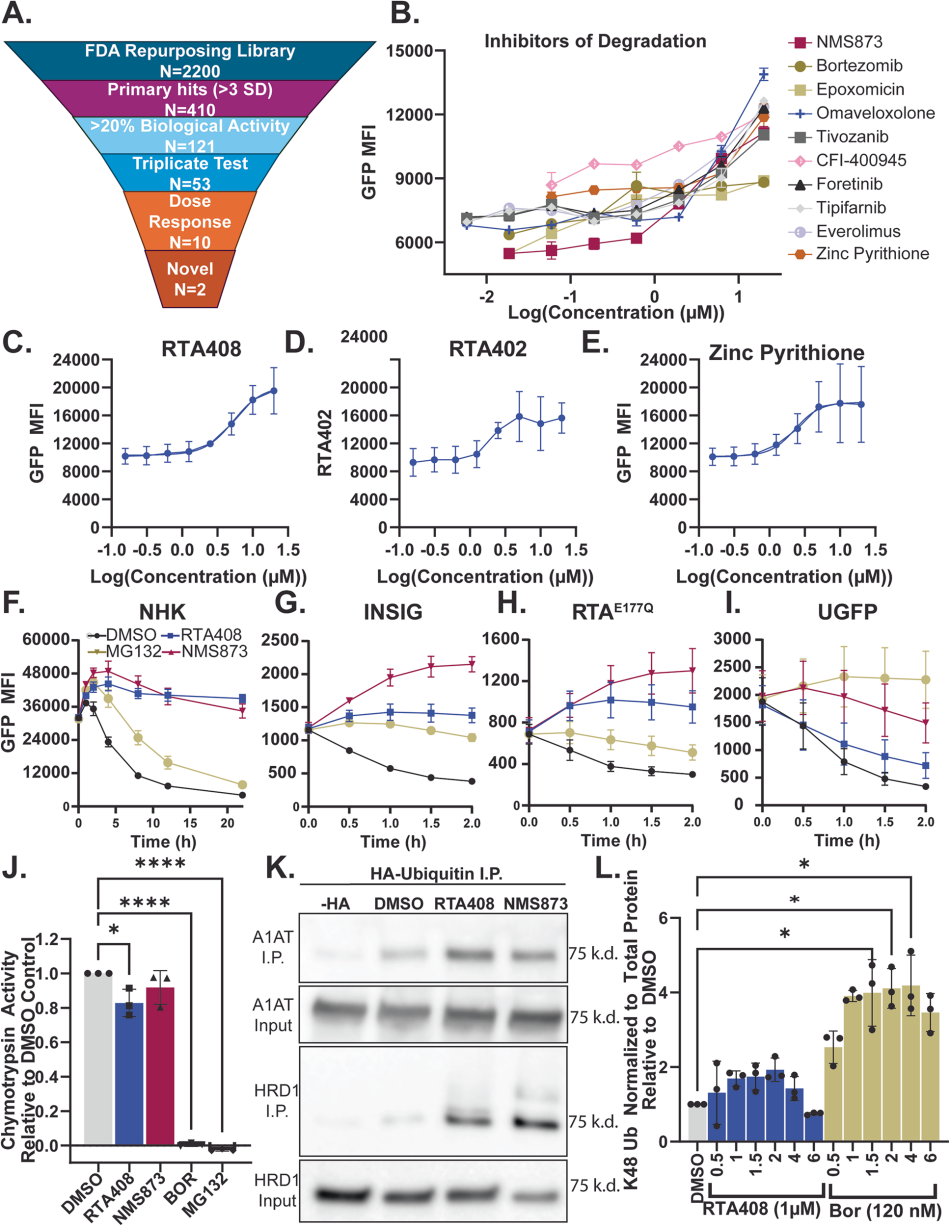
Identification of inhibitors of ERAD substrate degradation. **A** NHK-GFP HTS Triage Chart. **B** Dose response curve for steady state NHK-GFP degradation in K562 cells treated with small molecule inhibitors at 5.8 nM-20 µM at 4 h (*N* = 2 technical replicates). Dose response curve for inhibition of NHK-GFP steady state degradation by RTA408 (**C**) RTA402 (**D**) or zinc pyrithione (**E**) at 156 nM-20 µM at 4 h in K562 cells. Steady state degradation of NHK-GFP (**F**) INSIG-GFP (**G**) RTA^E177Q^-GFP (**H**) and uGFP (**I**) with DMSO or 10 µM RTA408, NMS873 or MG132 between 0–2 h in K562 cells. **J** Immunoprecipitation with HA-UB and detection of alpha-1 antitrypsin (A1AT) and HRD1 by immunoblot following 4 h steady state degradation with DMSO or 10 µM RTA408 or NMS873 in K562 cells. **K** Chymotrypsin activity measured by cell-based proteasome-Glo™ following 2 h treatment with DMSO, 1 µM RTA408, 10 µM NMS873, 120 nM BOR, or 10 µM MG132 in MM.1S. **L** Relative quantitation of immunoblotting for whole cell K48 ubiquitination with 1 µM RTA408 or 120 nM BOR treatment from 0.5–6 h in MM.1S cells. All steady state degradation experiments were performed with 20 µM emetine. *N* = 3 unless specified. Mean ± STDEV. Statistical analysis performed with ordinary one-way ANOVA with Dunnett’s multiple comparisons test (**J**)and Kruskal-Wallis test (**L**). *p ≤ 0.05, ****p ≤ 0.0001.

To identify ERAD inhibitors, we utilized a doxycycline-inducible GFP-tagged null Hong Kong variant of alpha-1 antitrypsin (NHK-GFP), a well-established ER-retained substrate that can be used to measure ERAD activity [25]. To develop a functional high-throughput screen (HTS), we optimized cell type, doxycycline induction, and flow cytometry parameters to measure the steady state degradation of NHK-GFP (Supplementary Fig. 1A–D). We screened 2,200 compounds from the FDA repurposing library (Fig. 1A). HTS software identified 121 primary hits, which were validated in triplicate testing [26]. Among these, 10 compounds exhibited an inhibitory concentration 50 (IC_50_) of ≤20 µM for NHK-GFP degradation (Fig. 1B and Supplementary Fig. 2A). Autofluorescent compounds that shifted the MFI independent of GFP positivity were removed (Supplementary Fig. 2B). Mechanistic prioritization revealed 3 compounds that are known ERAD inhibitors, including two PIs, bortezomib (BOR) and epoxomicin, and the VCP/p97 inhibitor NMS873 (Supplementary Fig. 2A). Of the remaining hits, five compounds have been previously tested in early phase clinical trials or pre-clinical studies in MM but have not been linked to ERAD activity [27–32] (Supplementary Fig. 2A). Given our goal of identifying novel ERAD inhibitors as therapeutics for MM, we selected the two ERAD inhibitors that have not been characterized in MM, omaveloxone (RTA408) and zinc pyrithione, for further validation.

Dose response curves for RTA408, bardoxolone methyl (RTA402-the parent compound for RTA408), zinc pyrithione, and two established VCP/p97 inhibitors (NMS873 and CB5083) showed an IC_50_ for NHK-GFP degradation in the low micromolar range (Fig. 1C–E and Supplementary Fig. 2C, D). We found that RTA408 extends the half-life of NHK-GFP from 4.8 h to >21 h as compared to a PI, MG132, which only increased the half-life of NHK-GFP to 10 h (Fig. 1F). Further analysis demonstrated dose-dependent inhibition of additional ERAD substrates, including the membrane protein INSIG and the non-glycosylated luminal substrate RTA^E177Q^ by both RTA408 and RTA402 (Fig. 1G, H and Supplementary Fig. 2E, F). There was minimal inhibition of degradation for the cytosolic protein unstable GFP (uGFP) by RTA408 at 10 µM or less (Fig. 1I and Supplementary Fig. 2G). Zinc pyrithione had limited inhibition of INSIG and RTA^E177Q^ degradation and was not pursued further (Supplementary Fig. 2E–G).

To assess the effects of RTA408 on the UPS, we evaluated chymotrypsin activity in the proteasome and found RTA408 has minimal effect compared to PIs (Fig. 1J). Similarly, NHK-GFP and HRD1 ubiquitination remained intact, indicating RTA408 doesn’t prevent the ubiquitination of ERAD substrates (Fig. 1K). RTA408 treatment did not significantly increase total K48-linked ubiquitination or overall ubiquitination levels, whereas BOR induced a pronounced accumulation (Fig. 1L and Supplementary Fig. 2H, I). Together, these findings suggest that RTA408 inhibits the degradation of both luminal and membrane ERAD substrates but has minimal effect on the degradation of select cytosolic proteins in a manner that is independent and distinct from PIs.

### Modulation of ERAD substrate degradation

**Fig. 2:**
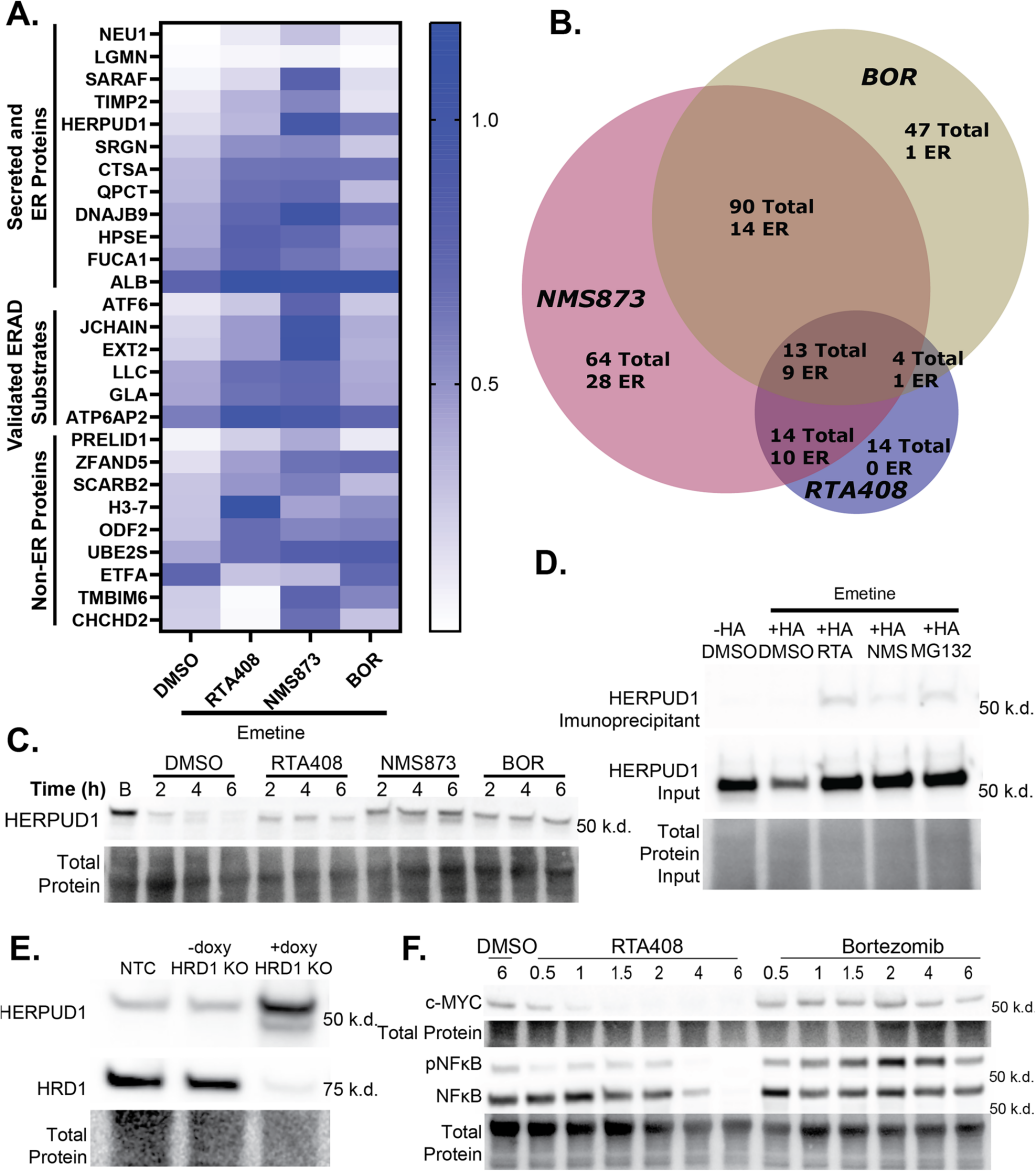
Proteomic analysis with ERAD inhibition. **A** Heatmap of relative protein abundance ratios normalized to DMSO control (no emetine) for steady state degradation (50 µM emetine) with DMSO, 1 µM RTA408, 10 µM NMS873, or 120 nM BOR in MM.1S at 4 h. *N* = 3. **B** Venn Diagram summarizing the number of proteins that had significantly altered steady state degradation (adjusted *p* ≤ 0.01) compared to DMSO+emetine control. **C** HERPUD1 immunoblot steady state degradation (50 µM emetine) with DMSO, 1 µM RTA408, 10 µM NMS873, or 120 nM BOR in MM.1S. **D** Immunoblot for HERPUD1 from immunoprecipitant (IP) or input from HA-tag K562 cells transduced with HA-ubiquitin and treated with DMSO, 10 µM RTA408, NMS873, or MG132. **E** Immunoblot of MM.1S transduced with non-targeting control (NTC) or doxycycline inducible HRD1 KO ± doxycycline. **F** Immunoblot of MM.1S cells treated with 1 µM RTA408 or 120 nM BOR for 0.5–6 h. Immunoblots are representative of *N* = 3–4 replicates.

To gain insight into the functional overlap of ERAD inhibition by RTA408, NMS873, and a PI, Bortezomib (BOR), we assessed their effects on steady state protein degradation by quantitative proteomics in MM (Fig. 2A, B and Supplementary Data S1). We observed a significant difference in the steady state degradation of 45 proteins with RTA408 (Fig. 2B). Of the 45 proteins, 44% are annotated in UniProt as ER proteins or contain an ER signal sequence, whereas only 33% and 16% of proteins altered by NMS873 or BOR are ER proteins, respectively [33]. Most of the targets of BOR were cytosolic proteins, consistent with its known function. Among the proteins shared between RTA408 and NMS873 treatments, 70% were ER proteins, including six validated ERAD substrates, such as lambda light chain and J-chain. Immunoblot analysis confirmed inhibition of lambda light chain degradation by RTA408 and NMS873 (Supplementary Fig. 3A, B), suggesting that RTA408 significantly alters ERAD substrate degradation.

In addition to known ERAD substates, our proteomic findings also identified 12 additional ER proteins stabilized by RTA408 and NMS873, which we hypothesized could be novel ERAD substrates. One such substrate is HERPUD1, which was stabilized by RTA408, NMS873 and BOR according to proteomic and immunoblot analysis (Fig. 2A–C and Supplementary Fig. 3C). To determine whether HERPUD1 undergoes degradation via the UPS, we performed HA-immunoprecipitation in K562 cells transduced with HA-ubiquitin and confirmed HERPUD1 ubiquitination, a key step required for ERAD degradation (Fig. 2D). Furthermore, we observed accumulation of HERPUD1 with ERAD deficiency by inducible HRD1 knockout in MM.1S (Fig. 2E and Supplementary Fig. 3D). Altogether these results suggest that RTA408 can be utilized as a tool to study ERAD in MM and identify novel ERAD substrates.

We also observed differential regulation of cytosolic substrates between RTA408 and BOR in MM cells. We detected a decrease in c-MYC levels, which is implicated in MM proliferation and progression, following RTA408 treatment whereas c-MYC levels were stabilized with BOR (Fig. 2F and Supplementary Fig. 3E) [17]. Similarly, BOR has been reported to inhibit IkB degradation, leading to increased phosphorylation and nuclear translocation of NFκB, a transcription factor that plays a key role in MM survival [18, 34]. We observed decreased NFκB phosphorylation with RTA408 treatment whereas BOR led to a moderate increase (Fig. 2F and Supplementary Fig. 3F). These findings highlight the differential effects of RTA408 and BOR on cytosolic protein regulation, underscoring the distinct consequences of alternative ERAD inhibition strategies on both ER and cytosolic protein homeostasis in MM.

### RTA408 cytotoxicity in MM cell lines

**Fig. 3:**
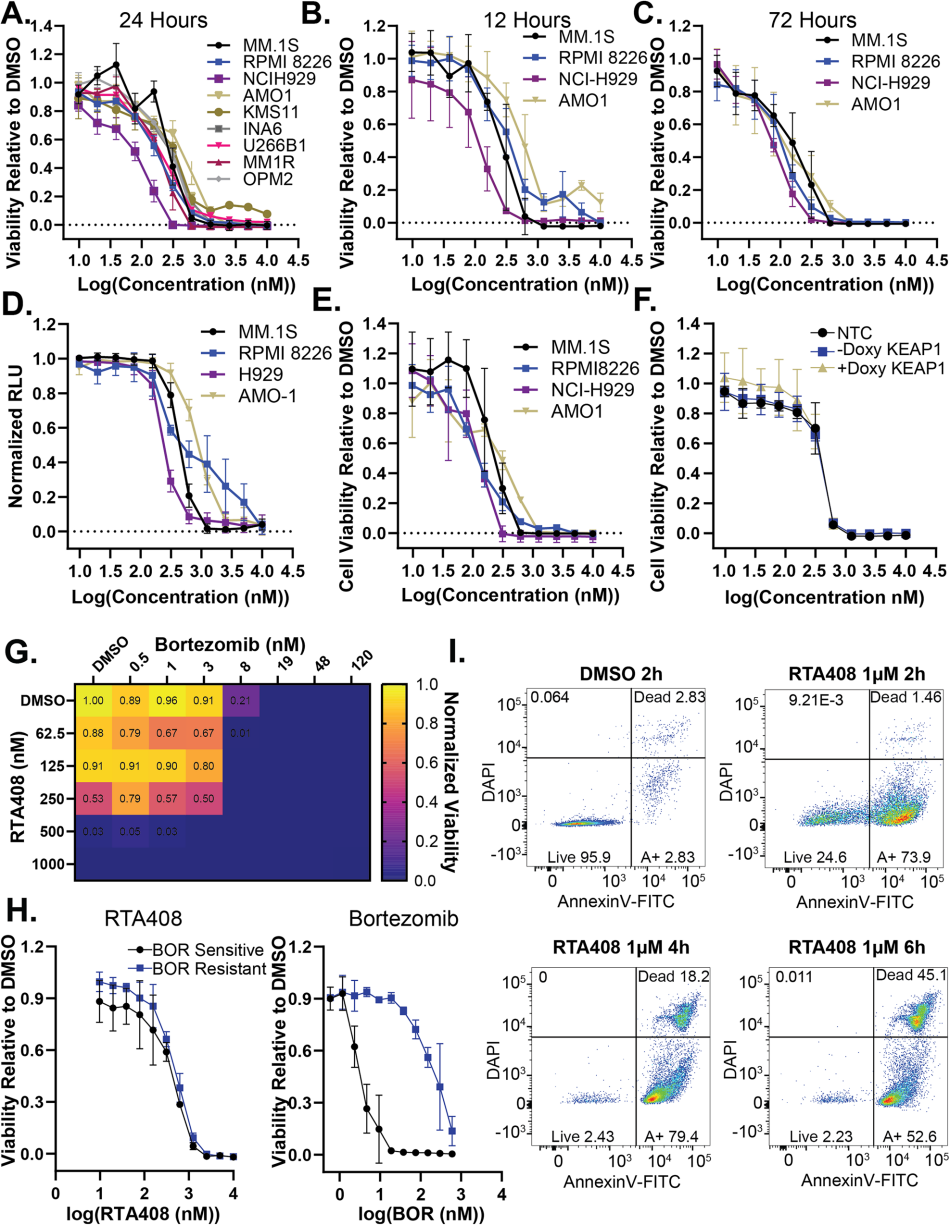
RTA408 cytotoxicity in Multiple Myeloma Cell Lines. MM cell line viability measured by CellTiter-Glo® following treatment with 10 nM-10 µM RTA408 for 24 h (**A**) 12 h (**B**) and 72 h (**C**). **D** Calcein AM uptake in MM cell lines treated with 10 nM-10 µM RTA408 for 24 h. **E** MM cell line viability measured by CellTiter-Glo® following treatment with 10 nM-10 µM RTA402 for 24 h. **F** MM cell line viability measured by CellTiter-Glo® in MM.1S cells with non-targeting control (NTC) or a doxycycline inducible KEAP1 KO ±doxycycline induction treated with 10 nM-10 µM RTA408 for 24 h. **G** Heatmap of mean viability measured by CellTiter-Glo® in MM.1S cells 24 h following Bortezomib 0.5-120 nM and RTA408 62.5-1000 nM cotreatment. Viability is normalized to the DMSO control. **H** Cell viability measured by CellTiter-Glo® in AMO1 Bortezomib sensitive and AMO1 bortezomib resistant cells treated with 10 nM-10 µM RTA408 or 0.6-600 nM Bortezomib for24h. **I** Representative flow cytometry plots of AnnexinV-FITC and DAPI staining in MM.1S cells treated with DMSO or 1 µM RTA408 at respective timepoints. *N* = 3. Mean ± STDEV.

We sought to test whether ERAD inhibition by RTA408 is cytotoxic to MM cells. RTA408 treatment decreased viability in nine MM cell lines with IC_50_ values ranging from 104-489 nM at 24 h (Fig. 3A and Supplementary Fig. 4A). Cytotoxicity occurred within 12 h with maximal effect by 72 h (Fig. 3B, C and Supplementary Fig. 4A). This was confirmed with alternative viability measurements using Calcein AM (Fig. 3D) and live/dead nuclear staining (Supplementary Fig. 4B). Similarly, RTA402 was also cytotoxic to MM (Fig. 3E). To determine whether RTA408-mediated cytotoxicity is dependent on its known target KEAP1, we performed inducible CRISPR-Cas9 knockout (KO) of KEAP1. KEAP1 KO did not alter RTA408 cytotoxicity (Fig. 3F and Supplementary Fig. 4C and 5A, B), suggesting that RTA408-induced MM cell death is independent of NRF2 activation.

Next, we evaluated the combinatorial effects of RTA408 with other MM therapies. Co-treatment with BOR did not enhance RTA408 cytotoxicity in MM.1S (Fig. 3G). RTA408 cytotoxicity did not correlate with BOR sensitivity across different cell lines (Supplementary Fig. 4B) and was not altered in a BOR-resistant AMO1 cell line (Fig. 3H and Supplementary Fig. 4A). In contrast, co-treatment with lenalidomide (Supplementary Fig. 4D) or dexamethasone (Supplementary Fig. 4E) exhibited a combinatorial effect. These data suggest that RTA408 cytotoxicity is independent of PI sensitivity.

Given the similar IC_50_ between 12 and 24 h treatment, we sought to determine the timing of cytotoxicity. We observed that RTA408 led to an early induction of apoptosis (1.5-2 h) measured by Annexin V positivity, followed by an increase in DAPI uptake within 4-6 h of treatment (Fig. 3I and Supplementary Fig. 4F–H). In contrast, the induction of apoptosis did not occur until after 6 h with NMS873 or BOR, suggesting RTA408 induces rapid cell death in MM cells.

### The activation of unfolded protein response (UPR) by ERAD inhibition

**Fig. 4:**
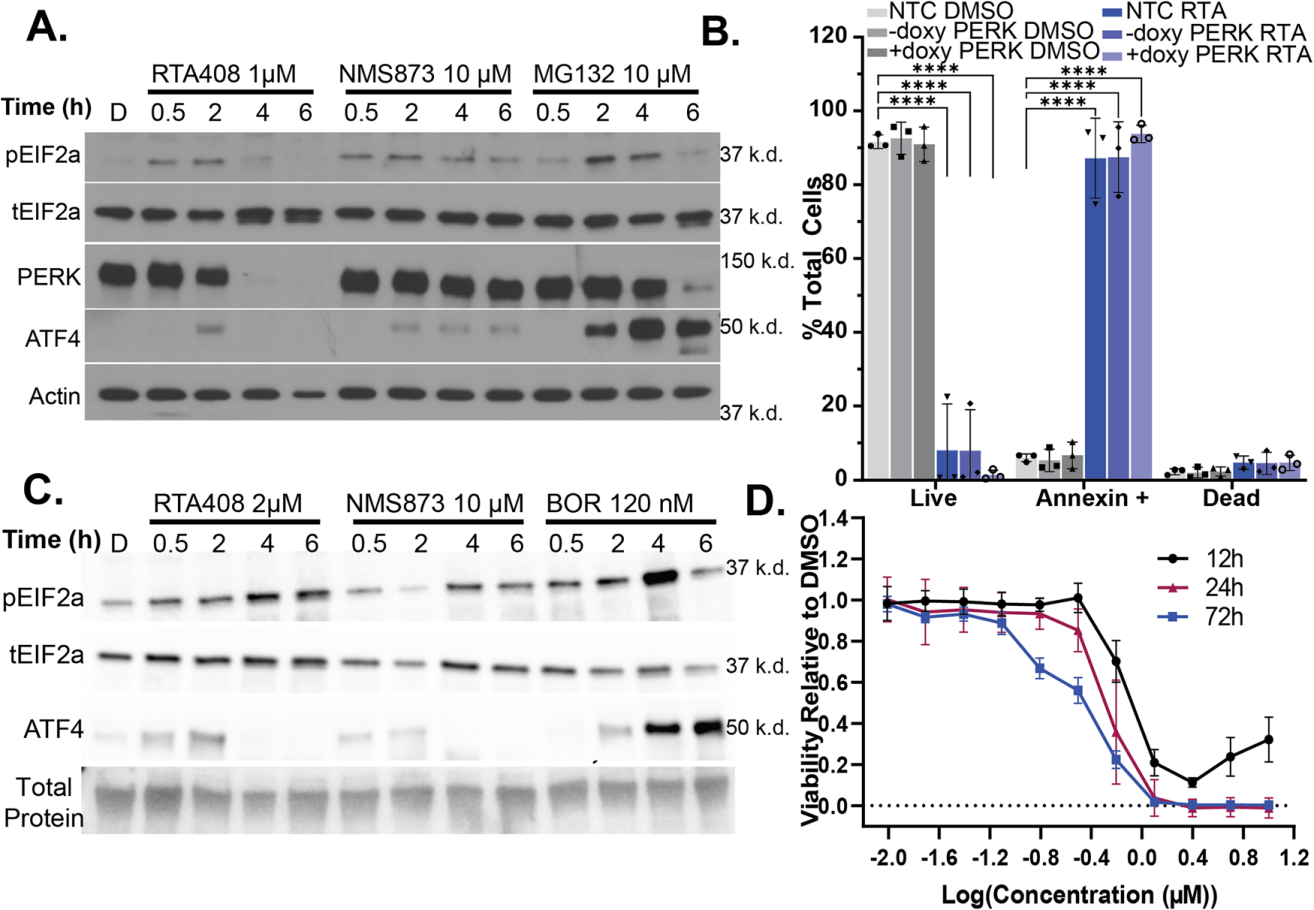
Cell death induced by ERAD inhibition is independent of UPR activation or immunoglobulin hypersecretion. **A** Immunoblot of pEIF2a, total EIF2a, PERK, ATF4, and β-Actin in MM.1 s treated with 1 µM RTA408, 10 µM NMS873, or 10 µM MG132 for 0.5–6 h. **B** Quantitation of live (AnnexinV-DAPI-), Annexin+ (AnnexinV+DAPI-) or dead (AnnexinV+DAPI + ) population 4 h in MM.1S cells transduced with non-targeting control, or doxycycline inducible PERK KO ± doxycycline. **C** Immunoblot of pEIF2a, total EIF2a, ATF4, and total protein quantitation in KMS12BM treated with 2 µM RTA408, 10 µM NMS873, or 120 nM BOR for 0.5–6 h. **D** KMS12BM cell line viability measured by CellTiter-Glo® following treatment with 10 nM-10 µM RTA408 for 12–72 h. Immunoblots are representative of *N* = 3–4 replicates. Mean ± STDEV. Statistical analysis performed with a two-way ANOVA with Tukey’s multiple comparison test. **** *p* < 0.0001.

Inhibition of ERAD has previously been associated with the rapid induction of ER stress and activation of the UPR. Indeed, treatment of different ERAD inhibitors led to a rapid induction of pEIF2α and accumulation of ATF4 in MM.1S and RPMI8226 cells (Fig. 4A and Supplementary Fig. 6A, B, Supplementary Fig. 7A, B). Increased mRNA expression of CHOP was detected within 2-4 h (Supplementary Figs. 6C and 7C). There was also an increase in IRE1α activation with an increased ratio of spliced/total XBP1 at 2 h (Supplementary Figs. 6D and 7D) without altering IRE1α levels (Supplementary Figs. 6E and 7E).

While induction of apoptosis has been associated with increased CHOP expression from UPR signaling, it has not been established that the UPR is directly responsible for early apoptotic signaling in response to ERAD inhibition in MM. To test this, we inhibited PERK by doxycycline inducible CRISPR-CAS9 KO or small molecule inhibitors of PERK (GSK2606414; G414) and pEIF2α activation (ISRIB). Despite moderately decreasing pEIF2α and ATF4 levels, inhibition of PERK or pEIF2a did not lead to a meaningful decrease in the annexin-positive population (Fig. 4B and Supplementary Fig. 8A–F). However, an important limitation to these studies is the incomplete ablation of pEIF2α or ATF4 accumulation. Next, we utilized treatment with emetine, which irreversibly inhibits transcription and protein translation, to evaluate if the induction of early apoptosis was dependent on the downstream transcription/translation of stress response proteins. We found that despite completely ablating ATF4 accumulation, emetine failed to rescue MM.1S cells from early apoptosis after RTA408 treatment (Supplementary Fig. 8G–I). These studies suggest that the early induction of apoptosis and cytotoxicity from RTA408 is independent of transcriptional and downstream responses of the UPR in MM.

It has been assumed that the activation of the UPR and ER stress responses is due to the secretory nature and high burden of paraprotein production of MM cells. To further evaluate the contribution of the UPR to RTA408-induced apoptosis, we evaluated KMS12BM cells, which lack paraprotein production at both the mRNA and protein levels [35]. We observed a rapid induction of pEIF2α and ATF4 accumulation and a time-dependent increase of apoptosis after RTA408 treatment in KMS12BM cells (Fig. 4C, D). These results suggest that RTA408-induced early apoptosis and cytotoxicity are independent of UPR signaling and paraprotein production in MM cells.

### Caspase 8 activation by ERAD inhibition

**Fig. 5:**
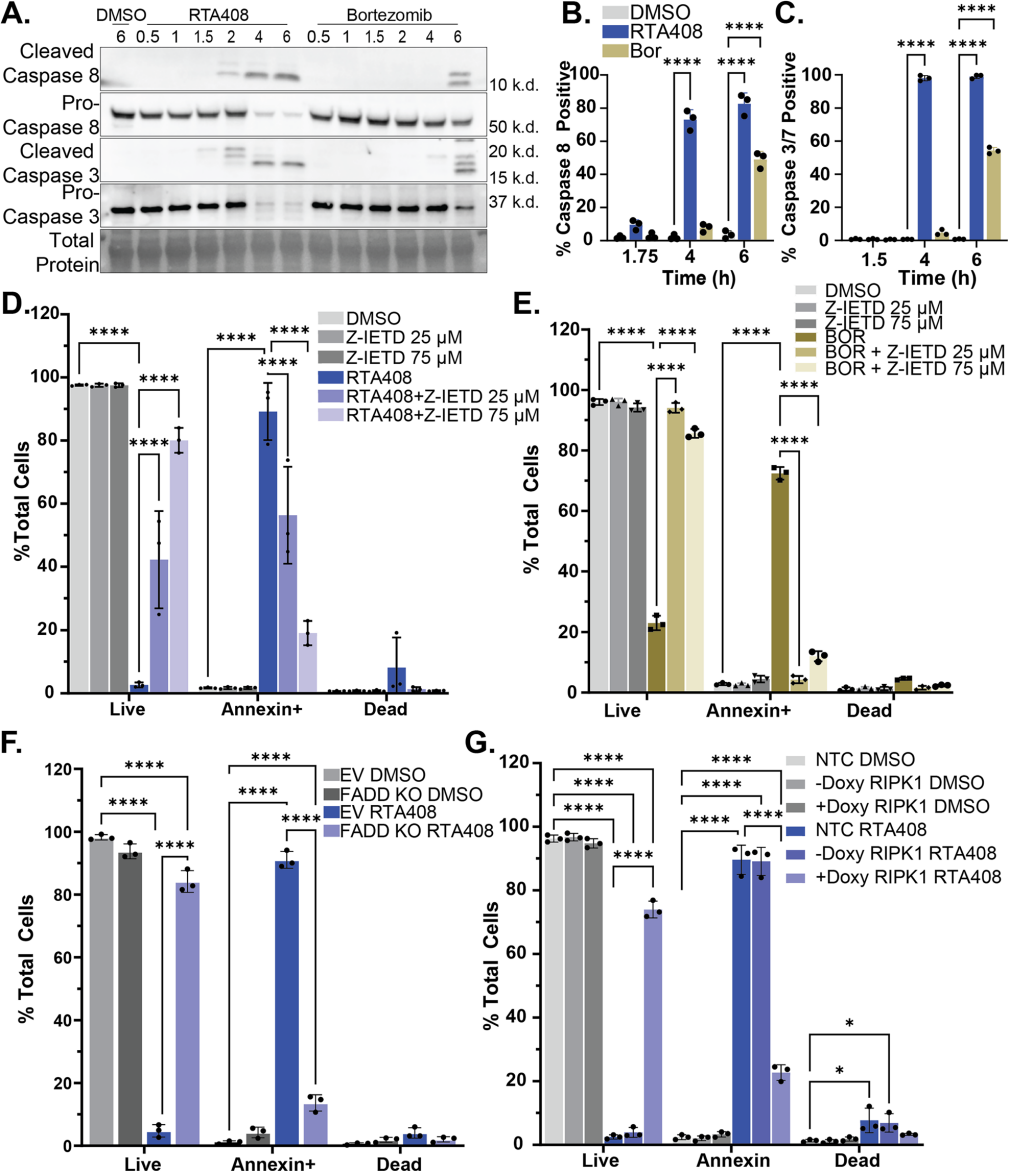
Pro-apoptotic signaling with ERAD inhibition. **A** Immunoblot of caspase 8 and 3 (cleaved and pro-forms) and total protein in MM.1 s treated with DMSO, 1 µM RTA408 or 120 nM BOR for 0.5-6 h. Flow cytometry quantitation of caspase 8 (**B**) or caspase 3/7 (**C**) activity in live MM.1S following DMSO, 1 µM RTA408 or 120 nM BOR for 1.5–6 h. Flow cytometry quantitation of live (AnnexinV-DAPI-), Annexin+ (AnnexinV+DAPI-) or dead (AnnexinV+DAPI + ) in MM.1S treated with 25–75 µM Z-IETD-FMK and DMSO or 1 µM RTA408 for 4 h (**D**) or 120 nM BOR for 6 h (**E**). Flow cytometry of Annexin V staining in MM.1S cells transduced with empty vector (EV), non-targeting control (NTC), or doxycycline inducible FADD KO (**F**) or RIPK1 KO (**G**) treated with DMSO or 1 µM RTA408 for 4 h. *N* = 3. Mean ± STDEV. Statistical analysis performed with a two-way ANOVA with Tukey’s multiple comparison test. *P < 0.05 ****p ≤ 0.0001.

We next evaluated activation of pro-apoptotic signaling following ERAD inhibition in MM as BOR has previously been associated with an induction of both intrinsic and extrinsic pro-apoptotic pathways [6, 36–39]. We observed rapid induction of cleaved caspase 8 and caspase 3 within 2 h of treatment with RTA408 and RTA402, which precedes activation at 6 h in BOR-treated MM.1 s cells (Fig. 5A and Supplementary Fig. 9A). This correlates with increased caspase 8 and caspase 3/7 activity with ERAD inhibition in both MM1S and RPMI8226 cells (Fig. 5B, C and Supplementary Fig. 9B; 10A, B). We also observed caspase 8 activation with ERAD deficiency by HRD1 KO (Supplementary Fig. 10C). Apoptosis from RTA408 was blocked by the addition of a pan-caspase inhibitor, Z-VAD-FMK, (Supplementary Fig. 9C) or a caspase 8 specific inhibitor, Z-IETD-FMK, in both MM.1S (Fig. 5D) and RPMI8226 (Supplementary Fig. 10D). We observed similar reversal of Annexin V positivity at 6 h with BOR treatment (Fig. 5E and Supplementary Fig. 9D). These results indicate that early apoptosis from ERAD inhibition in MM is mediated by caspase 8 activation.

### RTA408 induces aberrant lipid raft-dependent signaling

**Fig. 6:**
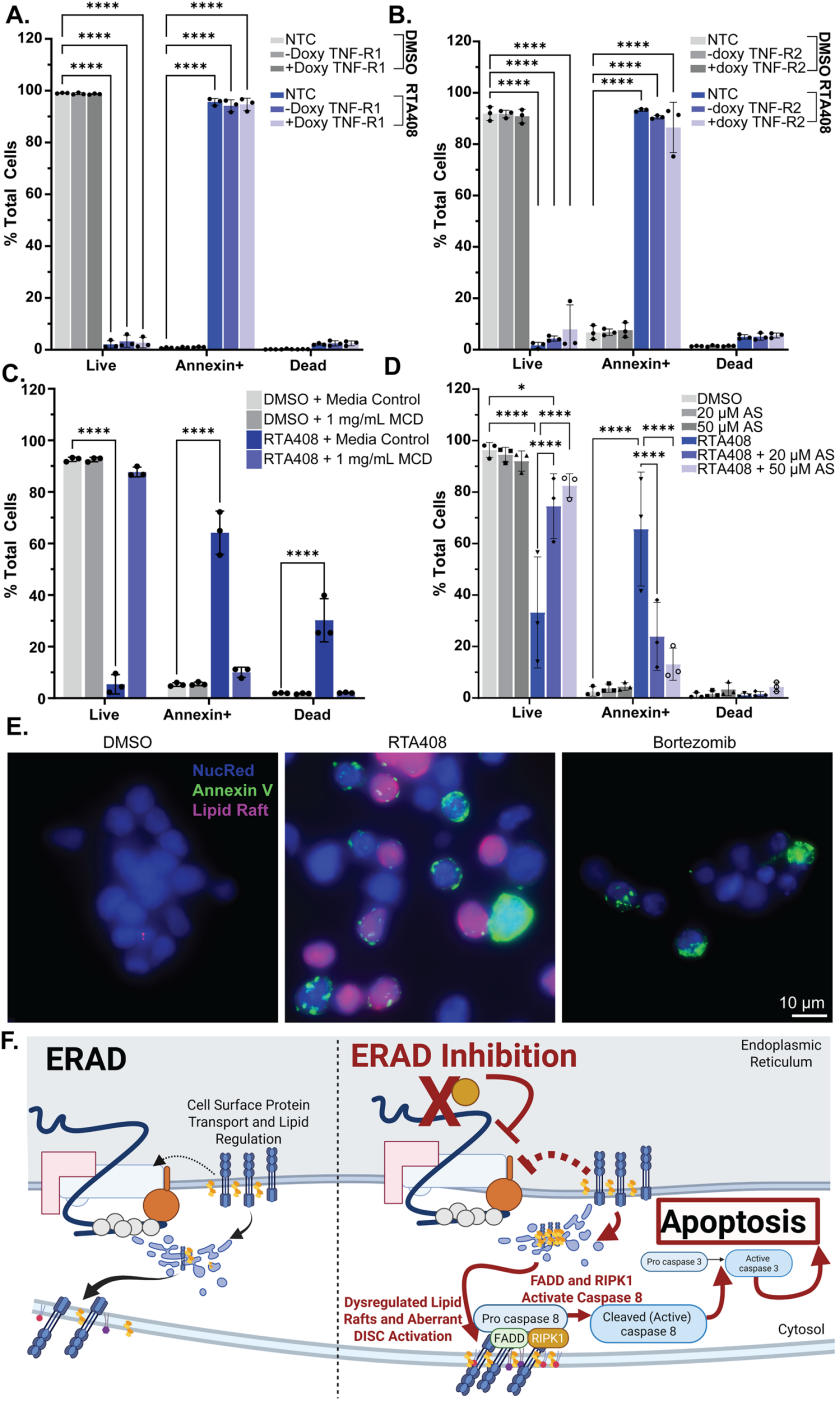
Lipid raft dependent activation of apoptosis with ERAD inhibition. Flow cytometry quantitation of live (AnnexinV-DAPI-), Annexin+ (AnnexinV+DAPI-) or dead (AnnexinV+DAPI + ) in MM.1S cells transduced with non-targeting control, or doxycycline inducible TNF-R1 (**A**) TNF-R2 (**B**) treated with DMSO or 1 µM RTA408 for 4 h. **C** Flow cytometry quantitation of Annexin V staining in MM.1S treated with 1 mg/mL MCD and DMSO or 1 µM RTA408 for 4 h. **D** Flow cytometry quantitation of Annexin V staining in MM.1S cells treated with DMSO or 1 µM RTA408 4 h and DMSO or 20-50 µM atorvastatin (AS) for 22 h. **E** Representative immunofluorescence images for MM.1S cells treated with DMSO, 1 µM RTA408, or 120 nM BOR for 2 h and stained for NucRed-Alexa647, Vybrant-Lipid Raft Label-Alexa555 (Lipid Raft), or AnnexinV-Alexa488. *N* = 3. **F** Schematic representing cell death signaling changes associated with ERAD inhibition by RTA408. Mean ± STDEV. Statistical analysis performed with a two-way ANOVA with Tukey’s multiple comparison test. ****p < 0.0001.

Caspase 8 signaling has been classically associated with activation of the extrinsic apoptotic pathway through the death-inducing signaling complex (DISC) (Supplementary Fig. 12H) [36, 40]. To determine whether DISC is required for ERAD inhibition-induced apoptosis, we performed CRISPR-Cas9 knockout of FADD and RIPK1, two essential DISC adapter proteins. FADD or RIPK1 KO prevented early apoptosis induced by RTA408 or BOR treatment (Fig. 5F, G and Supplementary Figs. 5C, D; 9E, F). Given the engagement of DISC adaptor proteins by cell death receptors, we next evaluated the regulation of FAS and tumor necrosis factor receptors (TNF-R1 and TNF-R2), which have been implicated in MM apoptotic signaling [41–43]. ERAD inhibition did not alter levels of cell death receptor proteins (Supplementary Fig. 11A) and inducible CRISPR-CAS9 KO of these receptors failed to rescue early apoptotic signaling (Fig. 6A, B, and Supplementary Figs. 5E–H; 11B–G), suggesting pro-apoptotic signaling is dependent on DISC but does not depend on the activation of individual cell death receptors [44].

Given that we were unable to identify a cell death receptor responsible for DISC activation, we evaluated non-canonical mechanisms for intracellular activation of caspase 8 [45–49]. Treatment with cathepsin inhibitors Gly-Phe-β-Naphthylamide (GPN) or Pepstatin A did not rescue RTA408-mediated apoptosis (Supplementary Fig. 12A, B) nor was early induction of apoptosis dependent on KEAP1 (Supplementary Fig. 12C). While IRE1α has been shown to activate RIPK1 [50], CRISPR-CAS9 knockout of IRE1α failed to restore the live population following RTA408 or BOR treatment (Supplementary Figs. 5I, and 12D, E). Finally, ERAD inhibition did not result in an increase in reactive oxygen species (ROS) production (Supplementary Fig. 12F, G).

To evaluate whether apoptotic induction is reliant on altered membrane-associated signaling, we treated cells with methyl-β-cyclodextrin (MCD), a compound known to deplete cholesterol from the plasma membrane, which disrupts both lipid rafts and DISC assembly [51]. Co-treatment with MCD restored the live cell population after RTA408 but not BOR treatment (Fig. 6C, Supplementary Figs. 10E, F, and 13A). Similarly, MCD treatment prevented caspase 8 cleavage and partially restored cell viability following 12 h of RTA408 and NMS873 treatment (Supplementary Fig. 13B, C). MCD did not affect NHK-GFP degradation and only partially rescued pEIF2α levels in MM.1S (Supplementary Fig. 13D–F), indicating that MCD did not impact the effects of RTA408 on ERAD activity, but its effects on pro-apoptotic signaling were ablated by MCD. This implies that RTA408, VCP/p97 inhibitors, and PIs induce pro-apoptotic caspase 8 signaling though distinct mechanisms and that the effects of RTA408 are dependent on membrane-associated DISC signaling.

Given that ERAD has been implicated in regulation of cholesterol synthesis and lipid rafts, we next investigated whether RTA408 altered intrinsic activation of the DISC through aberrant lipid raft organization. We found that inhibition of endogenous cholesterol synthesis with atorvastatin (AS) prevented the early induction of apoptosis with 2–4 h RTA408 treatment, without affecting pEIF2α activation (Fig. 6D and Supplementary Fig. 13G–J). Similarly, we observed increased staining of the lipid raft associated ganglioside GM1 by cholera toxin B staining after 2 h treatment with RTA408 (Fig. 6E). These data suggest that ERAD inhibition by RTA408 leads to altered lipid raft organization and aberrant DISC activation at the plasma membrane, resulting in the induction of pro-apoptotic signaling in MM (Fig. 6F).

### Cytotoxicity in MM models

**Fig. 7:**
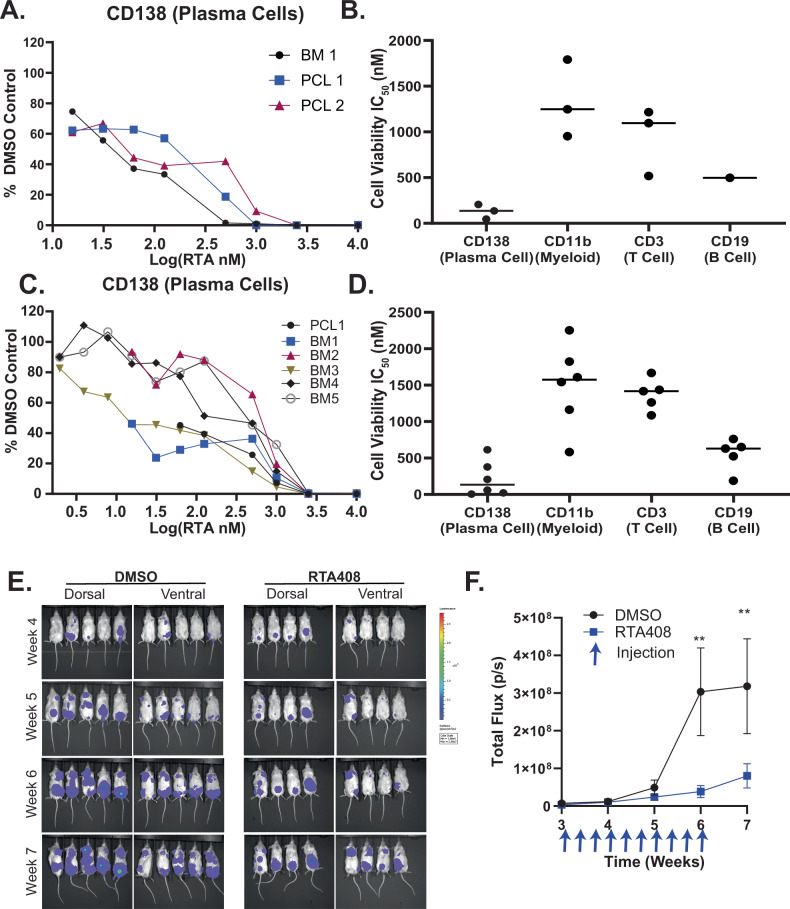
RTA408 is cytotoxic to primary malignant plasma cells and in vivo xenotransplant models of multiple myeloma. RTA408 Cytotoxicity in Primary Cells and In Vivo: Flow cytometry analysis of Annexin-DAPI- CD138 (**A**) and calculated IC50 in CD138, CD3, CD11b, and CD19 cells (**B**) following 60 h RTA408 in primary bone marrow (BM) or peripheral blood (PCL) cells from patients with relapsed refractory MM. Flow cytometry analysis of Annexin-DAPI- CD138 (**C**) and calculated IC50 in CD138, CD3, CD11b, and CD19 cells (**D**) cells following 36 h RTA408 in BM or PCL cells from patients with newly diagnosed MM. **E**, **F** Bioluminescent imaging and quantitation of total luciferin flux in NSG mice transplanted with 5e6 RPMI8226-luciferase cells. *N* = 5 (RTA408) and 6 (DMSO) for RPMI transplant. Mean ± STDEV. Statistical analysis performed with a two-way ANOVA with Tukey’s multiple comparison test. ** *p* < 0.01.

We evaluated the cytotoxicity of RTA408 in peripheral blood or bone marrow mononuclear cells collected from patients with R/R MM at the time of disease progression on PI-containing therapies. We observed maximal cytotoxicity of CD138^+^ plasma cells within 60 h of RTA408 (Fig. 7A, B). There was differential cytotoxicity when compared to non-malignant CD3^+^ T-cells and CD11^+^ myeloid cells (Fig. 7B and Supplementary Fig. 14A, B). However, intermediate toxicity was observed in the CD19⁺ B-cell population (Supplementary Fig. 14D). To assess whether there is a difference in susceptibility between treatment-naive and PI-resistant R/R MM, we tested RTA408 cytotoxicity in samples from newly diagnosed patients. While we observed similar dose response in CD138^+^ plasma cells, maximal toxicity occurred earlier within 36 h of treatment (Fig. 7C, D) in treatment naïve samples. Again, the non-malignant CD3^+^, CD11b^+^, and CD19^+^ (Fig. 7D and Supplementary Fig. 14C–E) cells were less sensitive to RTA408, suggesting a potential therapeutic index.

To evaluate the efficacy of RT408 in vivo, we utilized a bioluminescent xenograft model. In this model, RPMI8226 cells expressing luciferase were injected into sublethal irradiated NSG mice as previously described [23, 24]. Three weeks after injection, baseline imaging was performed, followed by intraperitoneal administration of RTA408 at 5 mg/kg every other day for three weeks. Bioluminescence imaging was performed on transplant recipients weekly until 7 weeks post-transplant. RTA408 treatment effectively prevented tumor growth (Fig. 7E, F and Supplementary Fig. 14F, G) as compared to the DMSO control. These data suggest that RTA408 is cytotoxic to primary malignant plasma cells and has in vivo anti-myeloma activity.

## Discussion

Despite its essential role in cellular function and survival, the regulatory mechanisms governing ERAD remain poorly understood [5]. A major challenge in studying ERAD is the limited availability of specific inhibitors that directly target ERAD activity. Current small-molecule modulators predominantly act on downstream pathways, such as VCP/p97 or the proteasome, which are not specific to ER protein degradation [1, 10]. In this study, we conducted a cell-based, high-throughput screen to identify small molecules that modulate ERAD activity. A pilot screen using the FDA-repurposing library successfully identified proteasome and VCP/p97 inhibitors as inhibitors of ERAD substrate degradation, validating the robustness of our screening platform. Our screen also identified RTA408 and its parent compound, RTA402, as novel ERAD inhibitors with distinct properties from VCP/p97 or proteasome inhibitors. This study establishes a valuable framework for identifying additional small-molecule modulators that regulate different ERAD components for research and potential therapeutic applications.

Several ERAD complex proteins have been identified as preferentially essential for MM cell survival, and ERAD inhibition has been proposed as a therapeutic strategy in MM [3, 52]. ERAD inhibition is thought to contribute the cytotoxicity of PIs, however, most patients develop PI resistance resulting in R/R MM [53]. PI resistance has been associated with altered protein homeostasis, prompting interest in targeting other ERAD components [53]. Here, we identified and characterized omaveloxolone (RTA408), an FDA-approved drug for Friedreich’s ataxia, as a novel ERAD inhibitor with potent cytotoxic activity against MM cells, independent of PI sensitivity. RTA408 induces rapid cell death via pro-apoptotic signaling, highlighting the vulnerability of MM cells to ERAD inhibition. Furthermore, RTA408 demonstrated cytotoxicity in primary malignant plasma cells from both newly diagnosed and R/R MM patients. Importantly, non-malignant T-cells and myeloid cells from the same patients were less affected, suggesting a potential therapeutic window for targeting ERAD in plasma cell neoplasms.

RTA408 treatment inhibited tumor growth in a xenograft MM model, supporting its in vivo anti-myeloma activity. Given that RTA408 and its parent compound, bardoxolone methyl, RTA402, have been previously tested in human clinical trials, they may be rapidly translated for evaluation in MM patients [14, 15]. In a prior phase I trial with RTA402, a patient with mantle cell lymphoma obtained a complete response and withdrew to receive a hematopoietic stem cell transplant, suggesting potential activity of ERAD inhibition in B-cell malignancies [54]. However, application in other cancer types will require careful consideration. While many ERAD proteins are required in plasma cell and B-cell neoplasms, early phase clinical trials with RTA408 or RTA402 revealed less activity in solid tumors and many proteins in ERAD pathway are largely dispensable in solid cancer and non-malignant cell lines [52, 54–56]. This highlights that ERAD regulation is cell-type specific, which, in combination with our findings, suggests that ERAD inhibition can be leveraged as a therapeutic strategy in malignancies like MM that are highly reliant on ERAD activity.

The proposed primary mechanism of action for RTA408 involves inhibiting the degradation of NRF2 via the KEAP1-CUL3 E3 ligase complex, thereby stabilizing NRF2 to induce an antioxidant response [57]. However, our data indicate that KEAP1 KO does not affect the cytotoxicity of RTA408, and pro-apoptotic signaling is independent of transcriptional responses, supporting that RTA408-induced cytotoxicity is independent of NRF2. While the precise molecular target and mechanism of ERAD inhibition by RTA408 remain to be investigated, our studies suggest that RTA408 prevents the degradation of luminal, membrane, and non-glycosylated ERAD substrates, while sparing many cytosolic proteins. Our proteomic analysis further highlights the specificity of RTA408 for ERAD substrates, and our mechanistic data suggest that RTA408 targets ERAD substrate degradation upstream of the proteasome, but future studies will focus on protein target identification.

Our findings also emphasize that targeting different ERAD components results in distinct effects on protein homeostasis and cytotoxicity in MM cells. PIs broadly inhibit the UPS, affecting both ERAD substrates and cytosolic proteins. In contrast, RTA408 selectively inhibits ERAD while sparing cytosolic proteins such as c-MYC, which may have implications for disease progression and relapse [17, 58]. These proteins have been shown to be upregulated in PI-resistant MM cells, raising the possibility that RTA408 may help overcome PI resistance through differential effects on cytosolic protein substrates.

While differential targeting of ER and cytosolic protein degradation may be promising for therapeutics in MM, additional studies are desirable for the clinical implementation of RTA408 or RTA402. Both compounds lead to NRF2 accumulation, which has been shown to improve MM survival and induce chemoresistance [59–61]. Target identification and chemical modification of RTA408 to allow for greater specificity towards ERAD inhibition could eliminate the secondary effects from NRF2 accumulation and enhance its anti-myeloma effects.

Our studies also discovered an unexpected mechanism by which ERAD inhibition induces apoptosis. The induction of the UPR, particularly through PERK/ATF4 signaling, has been linked to CHOP transcription and pro-apoptotic signaling in MM cells [62]. Previous studies have shown that ERAD inhibition in MM cells, via proteasome and VCP inhibitors, rapidly activates the UPR and triggers ER stress-induced apoptosis [3, 9, 12, 63]. However, our findings indicate that while these pathways are activated in MM cells, the early apoptotic response precedes CHOP accumulation and occurs independently of PERK signaling or translational stress responses. Moreover, ERAD-induced cytotoxicity is not dependent on the high secretory burden of MM cells, challenging the prevailing assumption that MM’s dependence on ERAD is primarily due to its highly active protein synthesis and secretion. This suggests that ERAD inhibition is not cytotoxic to MM cells merely by increasing ER stress from excessive misfolded protein accumulation in the ER.

Surprisingly, we found that disruption of ERAD by genetic deletion of HRD1 or chemical inhibition rapidly induces pro-apoptotic signaling mediated by caspase 8 in malignant plasma cells. A prior study suggested that PIs can induce aggregation of FADD, RIPK1, and caspase 8, leading to caspase-8-dependent apoptosis in colorectal cancer cells [39]. Whereas we find that RTA408 leads to membrane-dependent activation of the DISC complex, leading to caspase-8-dependent apoptosis. Caspase 8 activation by RTA408 can be reversed by methyl-β-cyclodextrin (MCD) treatment, suggesting that while both RTA408 and PIs activate caspase 8-dependent pro-apoptotic signaling, they do so through distinct mechanisms. These differences may explain why RTA408 does not exhibit additive cytotoxicity when combined with BOR yet remains effective against PI-resistant MM models.

Interestingly, ERAD inhibition-induced early apoptosis was independent of individual death receptors or non-canonical signaling, despite their known roles in DISC assembly and caspase 8 activation [39, 41–43, 45, 50, 64]. Instead, we found that RTA408-induced pro-apoptotic signaling is associated with altered lipid raft regulation. This raises the possibility that ERAD inhibition by RTA408 disrupts lipid homeostasis, membrane integration of death receptors, or simultaneously activates multiple death receptors [65–69]. Future studies should focus on elucidating the precise role of ERAD in lipid raft formation and DISC regulation in MM. Our findings highlight that the ERAD inhibitors identified from our high-throughput screen hold promise not only as potential therapeutics but also as tools to dissect cell type-specific ERAD regulation and downstream signaling.

## Supplementary information

Supplementary Methods and Figure Legends

Proteomics Supplementary Table

Supplementary Table 2 and 3: Materials and Oligonucleotides

Supplementary Figure 1

Supplementary Figure 2

Supplementary Figure 3

Supplementary Figure 4

Supplementary Figure 5

Supplementary Figure 6

Supplementary Figure 7

Supplementary Figure 8

Supplementary Figure 9

Supplementary Figure 10

Supplementary Figure 11

Supplementary Figure 12

Supplementary Figure 13

Supplementary Figure 14

Supp Fig 15 Uncropped Main Western Blot Images

Supp Fig 16 Uncropped Supplementary Western Blot Images

## Data Availability

The raw mass spectrometry proteomics data have been deposited and are available in the ProteomeXchange Consortium via the PRIDE partner repository with the dataset identifier PXD061058. Data that support this study are available upon request from the corresponding author, Qing Li.
