# Supplementary material for: Small molecule screening identifies cytotoxic endoplasmic reticulum-associated degradation inhibitors in multiple myeloma
Source: Cell Death Dis. 2026 Mar 9;17(1):303. doi: 10.1038/s41419-026-08526-2 (PMC13040074; doi:10.1038/s41419-026-08526-2)
Supplement: Supplementary file 1 — Supplementary Methods and Figure Legends [file 41419_2026_8526_MOESM1_ESM.docx]

**Supplementary Methods and Materials**

Supplies and Reagents: Supplemental Table 2 contains a comprehensive list of reagents containing the vendor and catalog numbers. A complete list of primers and guide RNA sequences can be found in Supplemental Table 3.

Plasmids: The GFP tagged null hong kong variant of alpha-1 antitrypsin (NHK) and transactivator plasmids were a gift from the Kopito lab. pLenti puro HA-Ubiquitin was a gift from Melina Fan (Addgene plasmid # 74218; http://n2t.net/addgene:74218 ; RRID:Addgene_74218). FgH1tUTG and FUCas9Cherry were a gift from Marco Herold (Addgene plasmid # 70183 and #70182; http://n2t.net/addgene:70183 and http://n2t.net/addgene:70182; RRIDs: Addgene70183 and 70182).

*Lentivirus Generation & Transduction:* Lentivirus was generated in 293T cells co-transfected with packaging plasmids pMD2.G and psPAX2 using FuGENE HD Transfection Reagent. CRISPR-CAS9 MM.1S cells were transduced by spinfection with lentivirus at 800 x g for 1.5h at 30 deg C followed by 1h incubation at 37C. K562 cells were transduced by infection with lentivirus at 800xg at RT for 1.75h.

*Small Molecule Screening:* NHK-GFP K562 cells were induced with doxycycline (0.75 µg/mL) for 16 hours. For screening, doxycycline was removed, and cells were plated on 384 well plates (Corning 3701) containing small molecules with 20 µM emetine in phenol red free RPMI 10% FBS. Compounds were pre-dispensed into 384 well plates using a Echo650 acoustic dispenser from Beckman Coulter. DMSO vehicle control was utilized with less than 0.5% DMSO. After four-hour incubation, cells were analyzed with automated flow cytometry (Biorad Ze5) with DAPI (1 µg/mL) dead cell exclusion. Primary hits were defined as >3 STDEV above DMSO control and were further prioritized as having >20% increase in MFI over DMSO control. Primary hits were validated in triplicate and were selected based on having at least three out of four instances with >3 STDEV above DMSO. Remaining compounds were then testes in 8-point concentration response curves in duplicate. Lead compounds with an IC_50_ of ≤20 µM were then tested in orthogonal assay to ensure target/mechanism specificity.

*Ubiquitin Immunoprecipitation:* K562 NHK-GFP cells expressing HA-Ubiquitin were induced with doxycycline (0.75 µg/mL) for 16 hours. Doxycycline was removed and cells were plated with 20 µM emetine with respective treatments. Following 4h treatment, cells were harvested and washed with PBS. Cells were lysed with IP Lysis buffer (Pierce) with cOmplete Mini Protease (Roche) and phosphatase inhibitor (Roche). Protein was quantified with the micro-BCA protein quantitation assay (Thermo Scientific). 500 µg total protein was loaded with HA-beads and immunoprecipation was performed with the HA-Tag IP/Co-IP kit per manufacturer’s instructions (Pierce) with 25 µL non-reducing sample buffer elution followed by the addition of β-mercaptoethanol. For immunoblot analysis 12.5 µL I.P and 10% of whole cell lysate input were loaded as a control.

*Immunoblotting:* Protein quantitation was performed with Qubit® Protein Assay (Thermo Fisher); 5-12 µg of protein was loaded per condition. Proteins were separated 4-20% Mini-Protean TGX gels (Biorad) with Tris/Glycine/SDS buffer (Biorad) and transferred to PVDF membranes. Total protein quantitation was performed with TotalStain Q (PVDF; Azure Biosystems) per manufacturer’s recommendations and membranes were blocked with 2% bovine serum albumin in Tris-buffered saline with Tween 20 (0.1%). Blots were assessed primary antibodies and secondary antibodies under conditions described in the key resources table.

*Proteasome Activity: MM.1S* cells were plated at 1.25e5 cells/mL and allowed to recover overnight. Cells were treated for 2h and immediately analyzed with the chymotrypsin-like proteasome Glo™ Cell Based Reagent per manufacturer’s instructions. Media only control was used as a blank and activity was normalized to DMSO control. Uncropped images of immunoblots from the main figures are available in supplemental figure 15 and supplemental figures are available in supplemental figure 16.

*Protein Digestion and TMT labeling:* MM.1S cells were treated with ERAD inhibitors for 4h in the presence of 50 uM emetine. Cells were harvested, washed with PBS at 4C, lysed with RIPA lysis buffer and protein was quantified with BCA assay. 100 µg total protein per condition were submitted to Proteomics Resource Facility at the University of Michigan for processing and mass spectrometry data acquisition. Briefly, upon reduction (5 mM DTT, for 30 min at 45 C) and alkylation (15 mM 2-chloroacetamide, for 30 min at room temperature) of cysteines in samples, the proteins were precipitated by adding 6 volumes of ice-cold acetone followed by overnight incubation at -20° C. The precipitate was spun down, and the pellet was allowed to air dry. The pellet was resuspended in 0.1M TEAB and overnight (~16 h) digestion with trypsin/Lys-C mix (1:50 protease:protein (for solution digestion) at 37° C was performed with constant mixing using a thermomixer. The TMT 16-plex reagents (ThermoFisher Scientific; A44521) were dissolved in 20 µl of anhydrous acetonitrile and labeling was performed by transferring the entire digest to TMT reagent vial and incubating at room temperature for 1 h. Reaction was quenched by adding 8 µl of 5% hydroxyl amine and further 15 min incubation. Labeled samples were mixed together, and dried using a vacufuge. An offline fractionation of the combined sample (~300 µg) into 12 fractions was performed using high pH reversed-phase chromatography (Zorbax 300Extend-C18, 2.1mm x 150 mm column on an Agilent 1260 Infinity II HPLC system). Fractions were dried and reconstituted in 9 µl of 0.1% formic acid/2% acetonitrile in preparation for LC-MS/MS analysis. Samples were labeled with TMT mass tag channels as described in TMT Infor (Supplemental Table 1).

*Liquid chromatography-mass spectrometry analysis*: To obtain superior quantitation accuracy, we employed multinotch-MS3, which minimizes the reporter ion ratio distortion resulting from fragmentation of co-isolated peptides during MS analysis ^1^. Orbitrap Ascend Tribrid equipped with FAIMS source (Thermo Fisher Scientific) and Vanquish Neo UHPLC was used to acquire the data. Two µl of the sample was resolved on an Easy-Spray PepMap Neo column (75 µm i.d. x 50 cm; Thermo Scientific) at the flow-rate of 300 nl/min using 0.1% formic acid/acetonitrile gradient system (3-19% acetonitrile in 72 min;19--29% acetonitrile in 28 min; 29-41% in 20 min followed by 10 min column wash at 95% acetonitrile and re-equilibration) and directly spray onto the mass spectrometer using EasySpray source (Thermo Fisher Scientific). FAIMS source was operated in standard resolution mode, with a nitrogen gas flow of 4.2 L/min, and inner and outer electrode temperature of 100 °C and dispersion voltage or -5000 V. Two compensation voltages (CVs) of -45 and -65 V, 1.5 seconds per CV, were employed to select ions that enter the mass spectrometer for MS1 scan and MS/MS cycles. Mass spectrometer was set to collect MS1 scan (Orbitrap; 400-1600 m/z; 120K resolution; AGC target of 100%; max IT in Auto) following which precursor ions with charge states of 2-6 were isolated by quadrupole mass filter at 0.7 m/z width and fragmented by collision induced dissociation in ion trap (NCE 30%; normalized AGC target of 100%; max IT 35 ms). For multinotch-MS3, top 10 precursors from each MS2 were fragmented by HCD followed by Orbitrap analysis (NCE 55; 45K resolution; normalized AGC target of 200%; max IT 200 ms, 100-500 m/z scan range).

*Cell Viability Assays:* For MM cell line viability assays cells were plated at 1.25e5 cells/mL and allowed to recover for at least 2h prior to treatment. Following 12-72h treatment, ATP-dependent cell viability was measured with CellTiter Glo® (Promega) per standard manufacturer’s protocol and normalized to DMSO control. For viability measurements by Calcein AM (Biotium), cells were stained with 0.1 µM of Calcein AM for 30 minutes at room temperature (RT) after 24h, followed by the addition of DAPI (1 µg/mL) and flow cytometric analysis (Bio-Rad ZE5). Live cells were defined by Calcein AM^+^ and DAPI^-^ and normalized to the DMSO control. At 48 h treatment, Readyprobes ™ Cell Viability Imaging Kit (Invitrogen) was utilized per manufacturer’s instructions with flow cytometric analysis (Bio-Rad ZE5).

*qPCR:* RNA was extracted using TRIzol Reagent (Invitrogen) following manufacturer guidelines. 6 uL of linear acrylamide was added after phase extraction to help precipitate RNA. RNA was reverse transcribed to cDNA using the High-Capacity cDNA Reverse Transcription Kit with Rnase Inhibitor (Applied Biosystems) following manufacturer guidelines. qPCR was performed (Applied Biosystems QuantStudio 3) using 10 ng of cDNA with SYBR Green PCR Master Mix (Applied Biosystems). qPCR primers are listed in Table 3.

*ROS Measurement:* MM.1S cells were loaded with 10 µM CM-H2DCFDA (Diluted 1:500 from 5 mM DMSO stock) in prewarmed HBSS for 30 min at 37C. Cells were washed with HBSS and resuspended in standard media under specified treatment conditions. After 2 hours, 200 uL of cells were collected, followed by the addition of DAPI (5 µg/mL) and flow cytometric analysis (Bio-Rad ZE5). For positive control, 150 µM H2O2 was added for 1h.

*Cell Surface Immunophenotyping:* At specified timepoints, 1.5e5 cells were collected and incubated with Human TruStain FcX™ (Biolegend) for 5 min at 4C, followed by the addition of cell surface antibodies for 10 min at 4C. Cells were washed and resuspended in Annexin Binding Buffer (Invitrogen) with Annexin V (5 µL) and DAPI (5 µg/mL). Samples were incubated for 15 min at RT followed by immediate analysis by flow cytometry (BD LSRFortessa).

*Annexin V Analysis of Early Apoptosis:* At respective timepoints, 1.5e5 cells were collected and washed with Hanks’ Balanced Salt Solution (GIBCO) with 3% bovine calf serum (Cytiva). Cells were resuspended in Annexin Binding Buffer (Invitrogen) with Annexin V (5 µL) and DAPI (5 µg/mL) and incubated for 15 min at RT followed by immediate analysis by flow cytometry (BD LSRFortessa). Uniform gating based on forward and side scatter for single cell events was performed.

*Caspase Activity Assay:* For caspase 8 activity assay, cells were treated for specified timepoints, at which time CaspaTag Caspase 8 Fluorescein reagent was added. Cells were incubated for 15 min at 37C under 5% CO2, at which time cells were harvested, washed with 1 mL Annexin Binding buffer and resuspended with Annexin V APC (5uL) and DAPI (5 µg/mL). 15 min at RT followed by immediate analysis by flow cytometry (BD LSRFortessa). Caspase 3/7 activity was measured with Cell Event Caspase-3/7 (Invitrogen) per manufacturer’s instructions with DAPI (1 µg/mL) dead cell exclusion. Caspase 3/7 activity was quantified by flow cytometry analysis (BD LSRFortessa) at specified timepoints.

*Generation of gRNA Plasmids:* gRNA sequences were designed using Benchling and cloned into the FgH1tUTG plasmid as previously described^2^. gRNA oligonucleotides are listed in Table 2. Successful cloning of gRNA sequence into FgH1tUTG was confirmed by Sanger sequencing (Azenta) using FgH1tUTGseq (5’- CAGACATACAAACTAAAGAAT-3’).

*Generation of CRISPR-Cas9 KO Lines:* MM.1S cells were transduced with FUCas9-Cherry. 72 hours after transduction, MM.1S cells were purified for Cas9 expression by fluorescence-activated cell sorting (SonyMA900) for mCherry expression to generate the MM.1S-Cas9 line. To generate MM.1S CRISPR KO lines, MM.1S-Cas9 cells were transduced with the generated gRNA plasmids. Cells were used for experiments at least 3-7 days after gRNA expression was induced by doxycycline (1 µg/mL). Confirmation of KO was completed by immunoblotting.

*Immunofluorescence:* 5e5 MM.1S cells were plated in 1 mL media and treated with ERAD inhibitors for 2 hours. Cells were collected and stained in 1 mL recombinant cholera toxin Subunit B Alexa Fluor ™ 555 (1 µ/mL), Annexin V FITC, and NucRed™ Live 647 ReadyProbes™ (2 drops) Reagent in complete media for 15 min at 4C. Cells were washed, fixed with 4% formaldehyde for 10 min at RT, and mounted with Prolong™ Gold Antifade Reagent. Fluorescence was imaged on a THUNDER Imaging System (Leica) using a 100X objective.

*Human Primary MM Samples:* Samples were cultured with RPMI 1640 growth media with 20% Fetal Bovine Serum (FBS; Fisher), 10 ng/mL recombinant human IL-6 and 1x Penicillin-Streptomycin-Glutamine (Gibco)

*Xenograft Transplant Imaging*: RPMI8226 cells were transduced with Luciferase-2A-GFP Lentivirus (GenTarget Inc) according to manufacturer guidelines. RPMI8226-Luc cells were purified by fluorescence-activated cell sorting (SonyMA900) based on GFP expression. 1e6 cells were transplanted into Starting from week 3 post-transplant, mice were administered RTA408 (5 mg/kg) or vehicle (10% DMSO in Corn Oil) intraperitoneally every other day until takedown. Bioluminescence imaging (Perkin Elmer IVIS Spectrum) was conducted every week starting 3 weeks post-transplant until takedown with intraperitoneal luciferin.

*Proteomic Analysis:* Proteome Discoverer (v3.0; Thermo Fisher) was used for data analysis. MS2 spectra were searched against SwissProt human protein database (v2023-09-13) using the following search parameters: MS1 and MS2 tolerance were set to 10 ppm and 0.6 Da, respectively; carbamidomethylation of cysteines (57.02146 Da) and TMT labeling of lysine and N-termini of peptides (304.2071 Da) were considered static modifications; oxidation of methionine (15.9949 Da) and deamidation of asparagine and glutamine (0.98401 Da) were considered variable. Identified proteins and peptides were filtered to retain only those that passed ≤1% FDR threshold. Quantitation was performed using high-quality MS3 spectra. The mass spectrometry proteomics data have been deposited to the ProteomeXchange Consortium via the PRIDE partner repository with the dataset identifier PXD061058"

*Statistical Analysis:* Z’ calculated as previously described^3^. IC_50_ was determined by variable slope-four parameter dose response curve fits. Statistical analyses used in these studies include t tests (2 samples), one-way ANOVA (>2 samples) or two-way anova (>2 samples with two parameters) in GraphPad Prism and are specified in the corresponding figure legends. *p≤0.05, ** p≤0.01, ***p≤0.001, ****p≤0.0001.

1 McAlister GC, Nusinow DP, Jedrychowski MP, Wühr M, Huttlin EL, Erickson BK *et al.* MultiNotch MS3 enables accurate, sensitive, and multiplexed detection of differential expression across cancer cell line proteomes. *Anal Chem* 2014; **86**: 7150–7158.

2 Aubrey BJ, Kelly GL, Kueh AJ, Brennan MS, O’Connor L, Milla L *et al.* An inducible lentiviral guide RNA platform enables the identification of tumor-essential genes and tumor-promoting mutations in vivo. *Cell Rep* 2015; **10**: 1422–1432.

3 Zhang JH, Chung TDY, Oldenburg KR. A Simple Statistical Parameter for Use in Evaluation and Validation of High Throughput Screening Assays. *J Biomol Screen* 1999; **4**: 67–73.

**Supplemental Figure Legends:**

Supplemental Figure 1: Validation of Screening Approach for ERAD Substrate Degradation. A. Representative flow cytometry plot for mean fluorescence of NHK-GFP in K562 cells treated with 20 µM emetine and DMSO or 10 µM NMS873 for 4h. -doxycyline is a negative control. B. Flow cytometry analysis of NHK-GFP degradation in live K562 cells in the presence of 20 µM emetine between 0-6h. N=3 C. Quantitation of mean fluorescence intensity of steady state degradation for NHK-GFP in K562 with DMSO or 10 µM NMS873 or MG132 at 4h. N=3 D. C. Quantitation of mean fluorescence intensity of steady state degradation for NHK-GFP in K562 with DMSO or 10 µM NMS873 at 4h by automated flow cytometry in a 384 well plate used to calculate Z’. N=16-32 technical replicates. Mean±STDEV. Statistical analysis performed with a one-way ANOVA with Dunnett’s multiple comparison test. *p≤0.05, ***p≤0.001

Supplemental Figure 2: Validation of FDA Repurposing Library Hits. A. Table summarizing the top 10 hits from the FDA repurposing library with IC_50_, relative activity of NMS873 control, and hillslope estimated by variable slope-four parameter dose response curve based on curve represented in Figure 1A. B-D. Dose response curve for inhibition of NHK-GFP steady state degradation by PHA-665752(B), NMS873 (C), or CB5083 (E) at 156 nM-20 µM at 4h in K562 cells. E-G. Steady state degradation INSIG-GFP (E), RTA^E177Q^-GFP (F), and uGFP (G) with DMSO or 156 nM-20 µM RTA408, RTA402, or Zinc Pyrithione at 1h in K562 cells. H. Representative immunoblot of K48, total ubiquitin and total protein quantitation in MM.1S cells following treatment with DMSO, 1 µM RTA408, 10 µM NMS873, or 120 nM BOR for 0.5-6 h. I. Relative quantitation of total ubiquitin normalized for total protein quantitation from immunoblots in H. N=3. Mean±STDEV. Statistical analysis performed with a Kruskal-Wallis test with Dunn’s multiple comparisons. *p≤0.05.

Supplemental Figure 3. Validation of Proteomic Analysis. A-B. Immunoblot (A) and relative quantitation (B) for lambda light chain steady state degradation (50 µM emetine) with DMSO, 1 µM RTA408, 10 µM NMS873, or 10 µM MG132 in MM.1S. C-E. Relative quantitation for immunoblot analysis of steady state degradation of HERPUD1 (C), HERPUD1 in HRD1 KO MM.1s (D), c-MYC(E), or pNFKB(F). Statistical analysis with a two-way ANOVA with Tukey’s multiple comparisons (B-C; DMSO 0h was excluded from statistical analysis) or one-way ANOVA with Dunnett’s multiple comparisons (D-F). N=3 for CMYC and HRD1 blots. N=4 for NFkB, Lambda light chain, and HERPUD1 blots. * p≤0.05, **p≤0.01, ***p≤0.001, **** p≤0.0001

Supplemental Figure 4. RTA408 cytotoxicity in MM cells. A. Summary of IC_50_ for RTA408 cytotoxicity between 12-72h determined by CellTiter-Glo or Calcein AM staining. B. Cell viability determined by live/dead staining (Invitrogen) following 48 h treatment with 10 nM-10 µM RTA408, NMS873, or MG132 in MM.1S and RPMI8226 cells. C-D. Heatmap with viability measured by CellTiter-Glo® in MM.1S cells 24h following 39nM-5 µM lenalidomide (C) or 39nM-5 µM dexamethasone (D) and RTA408 62.5-1000 nM cotreatment at 72h. Viability is normalized to DMSO control. F-H. Quantitation of live (AnnexinV-DAPI-), Annexin+ (AnnexinV+DAPI-) or dead (AnnexinV+DAPI+) population at 2(F), 4 (G), or 6h (H) in MM.1S cells. N=3. Statistical Analysis by 2-way ANOVA with Dunnett’s multiple comparison tests. **p≤0.01, ***p≤0.001, **** p≤0.0001.

Supplemental Figure 5. Validation of CRISPR-CAS9 Inducible KO. Representative immunoblot of NTC, and + or – doxycycline with sgKEAP1 #1 (A) and sgKEAP1 KO #2 (B), sgFADD (C), sgRIPK1(D), sgTNF-R1 (E), sgTNF-R2 (F), sgDR4 (G) or sgDR5 (H), or sgIRE1α (I) in MM.1S cells.

Supplemental Figure 6. UPR Activation in MM.1S. A. Relative quantitation of PERK, pEIF2A, and ATF4 immunoblots for MM.1S treated with DMSO, 1 µM RTA408, 10 µM NMS873, or 10 µM MG132 in MM.1S for 0.5-6h. B. Immunoblot of pEIF2a, total EIF2a, PERK, ATF4, and β-Actin in MM.1s treated with 1 µM RTA402, 10 µM CB5083, or 120 nM BOR for 0.5-6h. C-D. Relative qPCR quantitation of CHOP (C), spliced or total XBP1 (D) relative to β-Actin in MM.1S treated with 1 µM RTA408, 10 µM NMS873, or 10 µM MG132 for 2-6h normalized to DMSO 6h control. N=4 for XBPS and XBPT qPCR. E. Immunoblot of IRE1α, and β-Actin in MM.1s treated with 1 µM RTA408, 10 µM NMS873, or 10 µM MG132 for 0.5-6h. N=3 unless specified. Mean±STDEV. No statistical analysis performed (A), statistical analysis with Kruskal-Wallis test with Dunn’s multiple comparisons test (C-D). *p≤0.05, **p≤0.01, ***p≤0.001

Supplemental Figure 7. UPR Activation in RPMI8226. A-B. Relative quantitation (A) and Representative immunoblots (B) of PERK, pEIF2A, and ATF4 immunoblots for RPMI8226 treated with DMSO, 1 µM RTA408, 10 µM NMS873, or 10 µM MG132 for 0.5-6h. C-D. Relative qPCR quantitation of CHOP (C), spliced or total XBP1 (D) relative to β-Actin in RPMI8226 treated with 1 µM RTA408, 10 µM NMS873, or 10 µM MG132 for 2-6h normalized to DMSO 6h control. E. Immunoblot of IRE1α, and β-Actin in RPMI8226 treated with 1 µM RTA408, 10 µM NMS873, or 10 µM MG132 for 0.5-6h. N=3. Mean±STDEV. No statistical analysis performed (A), statistical analysis with Kruskal-Wallis test with Dunn’s multiple comparisons test (C-D)*p≤0.05, **p≤0.01

Supplemental Figure 8. PERK Inhibition with ERAD Inhibition. A-B. Immunoblot (A) and relative quantitation (B) for pEIF2a, total EIF2a, PERK, and total protein in in MM.1S cells transduced with non-targeting control, or doxycycline inducible PERK KO (+or-doxycycline) treated with RTA408 1 µM for 2h. C and E. Quantitation of live (AnnexinV-DAPI-), Annexin+ (AnnexinV+DAPI-) or dead (AnnexinV+DAPI+) populations by flow cytometry in MM.1S cells treated with DMSO, RTA408 1 µM, 250 nM or 1 µM ISRIB (C) or GSK2606414 (G414) (E) for 4h. D. Representative immunoblot of ATF4 and total protein in MM.1S treated with with DMSO, RTA408 1 µM, 250 nM or 1 µM ISRIB for 2h. F. Representative immunoblot of pEIF2a and tEIF2a in MM.1S treated with with DMSO, RTA408 1 µM, 250 nM or 1 µM GSK2606414 (G414) for 0.5-2h. 1 µM Tunicamycin (TUN) at 6h was used as a positive control for G414 mediated inhibition of PERK signaling. G. Quantitation of Annexin V staining by flow cytometry in MM.1S cells treated with DMSO, RTA408 1 µM and H2O control or 50 µM emetine (labelled E) for 4h. H-I. Representative immunoblot (H) and relative quantitation (I) of pEIF2a, total EIF2a, ATF4, and total protein in in MM.1S at 2h with DMSO or RTA408 1 µM combined with H2O control or 50 µM emetine. N=3-4. *p≤0.05, ****p≤0.0001

Supplemental Figure 9: Pro-Apoptotic Signaling with ERAD Inhibition in MM.1S. A. Immunoblot of caspase 8 and 3 (cleaved and pro-forms) and total protein in MM.1s treated with DMSO, 1 µM RTA402, 10 µM CB5083 or 120 nM BOR for 0.5-6h.. B. Flow cytometry quantitation of caspase 3/7 activity in live MM.1S following DMSO, 1 µM RTA408 or RTA402, 10 µM CB5083 or NMS873, or 120 nM BOR for 1.5-6h. DMSO, RTA408, and BOR data are also represented in figure 5C. C-D. Flow cytometry quantitation of live (AnnexinV-DAPI-), Annexin+ (AnnexinV+DAPI-) or dead (AnnexinV+DAPI+) in MM.1S treated with 20-50 µM Z-VAD-FMK (C-D) and DMSO, 1 µM RTA408 for 4h (C), or 120 nM BOR for 6h (D). E-F. Flow cytometry annexin V staining in MM.1S cells transduced with empty vector (EV), non-targeting control (NTC), or doxycycline inducible FADD KO (E) or RIPK1 KO (F) treated with DMSO or 120 nM BOR for 6h. N=3. Mean±STDEV. Statistical analysis performed with a two-way ANOVA with Tukey’s multiple comparison test. ****p≤0.0001

Supplemental Figure 10: Pro-Apoptotic Signaling with ERAD Inhibition. A-B. Flow cytometry quantitation of caspase 8 (A) or caspase 3/7 (B) activity in live RPMI8226 following DMSO, 1 µM RTA408 or RTA402, 10 µM CB5083 or NMS873, or 120 nM BOR for 1.5-6h. C. Representative immunoblot of pro-caspase 8, cleaved caspase 8, and total protein quantitation in MM.1S transduced with non-targeting control (NTC) and doxycycline inducible HRD1 CRISPR-CAS9 KO (+ or – doxycycline). D. Flow cytometry quantitation of live (AnnexinV-DAPI-), Annexin+ (AnnexinV+DAPI-) or dead (AnnexinV+DAPI+) in RPMI8226 treated with 25-75 µM Z-IETD-FMK and DMSO or 1 µM RTA408 4h. E-F. Flow cytometry quantitation of Annexin V staining in RPMI8226 treated with 1 mg/mL MCD or media control and DMSO, 1 µM RTA408 for 4h (E) or 120 nM BOR for 6h (F). N=3. Mean±STDEV. Statistical analysis performed with a two-way ANOVA with Tukey’s multiple comparison test. *p p≤0.05, **p≤0.01, ****p≤0.0001

Supplemental Figure 11: Investigation of Cell Death Receptor Mediated Activation of Caspase 8. A. Immunoblot of FAS, FADD, BID, RIPK1, TNF-R1, TNF-R2 and total protein levels in MM.1S treated with DMSO, 1 µM RTA408, or 120 nM BOR for 0.5-6h. B. Flow cytometry quantitation of live (AnnexinV-DAPI-), Annexin+ (AnnexinV+DAPI-) or dead (AnnexinV+DAPI+) MM.1S cells transduced with non-targeting control, or doxycycline inducible FAS treated with DMSO or 1 µM RTA408 for 4h. C-E. Flow cytometry quantitation of AnnexinV staining in MM.1S cells transduced with non-targeting control, or doxycycline inducible FAS (C), TNF-R1 (D), TNF-R2 (E), (+or-doxy) treated with DMSO or 120 nM BOR for 6h. F-G. Flow cytometry quantitation of Annexin V staining in MM.1S cells transduced with non-targeting control, or doxycycline inducible DR4 or DR5 KO (+or-doxy) treated with DMSO or 1 µM RTA408 for 4h (F) or DMSO or 120 nM BOR for 6h (G). N=3-4. Mean±STDEV. Statistical analysis performed with a two-way ANOVA with Tukey’s multiple comparison test. **p≤0.01, ****p≤0.0001.

Supplemental Figure 12. Evaluation of Intracellular Pathways Implicated with Caspase 8 Activation. A-B. Flow cytometry quantitation of live (AnnexinV-DAPI-), Annexin+ (AnnexinV+DAPI-) or dead (AnnexinV+DAPI+) in MM.1S cells treated with Gly-Phe-β-Naphthylamide (GPN-A) or Pepstatin A (PepA-B) in combination with DMSO or 1 µM RTA408 for 4h. C-E. Flow cytometry quantitation of Annexin V staining in MM.1S cells transduced with non-targeting control, or doxycycline inducible KEAP1 treated with DMSO or 1 µM RTA408 for 4h (C) or IRE1α treated DMSO, 1 µM RTA408 for 4h (D) or 120 nM BOR (E) for 6h. F-G. Representative histogram (F) and flow cytometry quantitation (G) of CM-H2DCFDA mean fluorescence intensity (MFI) in live MM.1S cells. H. Graphical summary of caspase 8 dependent pro-apoptotic signaling implicated with ERAD inhibition. N=3. Mean±STDEV statistical analysis performed with a two-way ANOVA with Tukey’s multiple comparison test. ****p≤0.0001.

Supplement 13: Lipid Raft Dependent Activation of Caspase 8 in MM.1S. A. Flow cytometry quantitation of Annexin V staining in MM.1S treated with 1 mg/mL MCD or media control and DMSO or 120 nM BOR for 6h. B. Representative immunoblot of caspase 8 and 3 (cleaved and pro-forms) and total protein in MM.1s treated with DMSO, 1 µM RTA408 or 120 nM BOR and media control or 1 mg/mL MCD for 4-6h. C. MM cell line viability measured by CellTiter-Glo® following treatment with 1 µM RTA408, 10 µM NMS873 or 120 nM BOR and media control or 1 mg/mL MCD for 12h. D-E. Representative immunoblot (D) and relative quantitation (E) of phospho-EIF2α or total EIF2 α with DMSO, 1 µM RTA408 or 120 nM BOR and media control or 1 mg/mL MCD for 2h. F. Steady state degradation of NHK-GFP in K562 with 10 µM RTA408, NMS873 or MG132 and media control or 1 mg/mL MCD for 4h. G-H. Flow cytometry quantitation of Annexin V staining in MM.1S cells treated with DMSO or 20-50 µM atorvastatin (AS) and DMSO or 1 µM RTA408 for 4h (G-shown in main figure) or 120 nM BOR for 6h (H). I-J. Representative immunoblot (I) and relative quantitation (J) of phospho-EIF2α or total EIF2α with DMSO, 1 µM RTA408 or 120 nM BOR and DMSO or 20-50 µM atorvastatin (AS) for 2h. N=3-5. Mean±STDEV. Statistical analysis performed with a two-way ANOVA with Dunnett’s multiple comparison test (E) or Tukey’s multiple comparison tests. *p≤0.05, **p≤0.01, *** p≤0.001, p****p≤0.0001.

Supplemental Figure 14: RTA408 Cytotoxicity in Primary Cells and In Vivo. A. Representative flow cytometry gating of CD138+,CD3+ and Live (Annexin-DAPI-), Annexin+ (Annexin+DAPI-), and Dead (DAPI+) cells. B-C. Calculation of IC50 for cytotoxicity in different cell populations R/R (B) and newly diagnosed (C) samples. D-E. Flow cytometry analysis of Annexin-DAPI- CD19 cells following RTA408 treatment for 60h in R/R (D) and 36h (E) in newly diagnosed MM from primary bone marrow (BM) or peripheral blood (PCL) cells from patients with relapsed refractory MM. F. Week 3, pretreatment bioluminescent imaging in RPMI8226 NSG mice. G. Bioluminescent imaging from a pilot cohort of RPMI8226 NSG mice; left treated with RTA408 5 mg/kg; right DMSO control. Center mouse not included in treatment schema.

Supplemental Figure 15: Uncropped images from representative immunoblots in main text figures.

Supplemental Figure 16: Uncropped images from representative immunoblots in supplemental figures.
