# Supplementary figures and images for: Small molecule screening identifies cytotoxic endoplasmic reticulum-associated degradation inhibitors in multiple myeloma
Source: Cell Death Dis. 2026 Mar 9;17(1):303. doi: 10.1038/s41419-026-08526-2 (PMC13040074; doi:10.1038/s41419-026-08526-2)

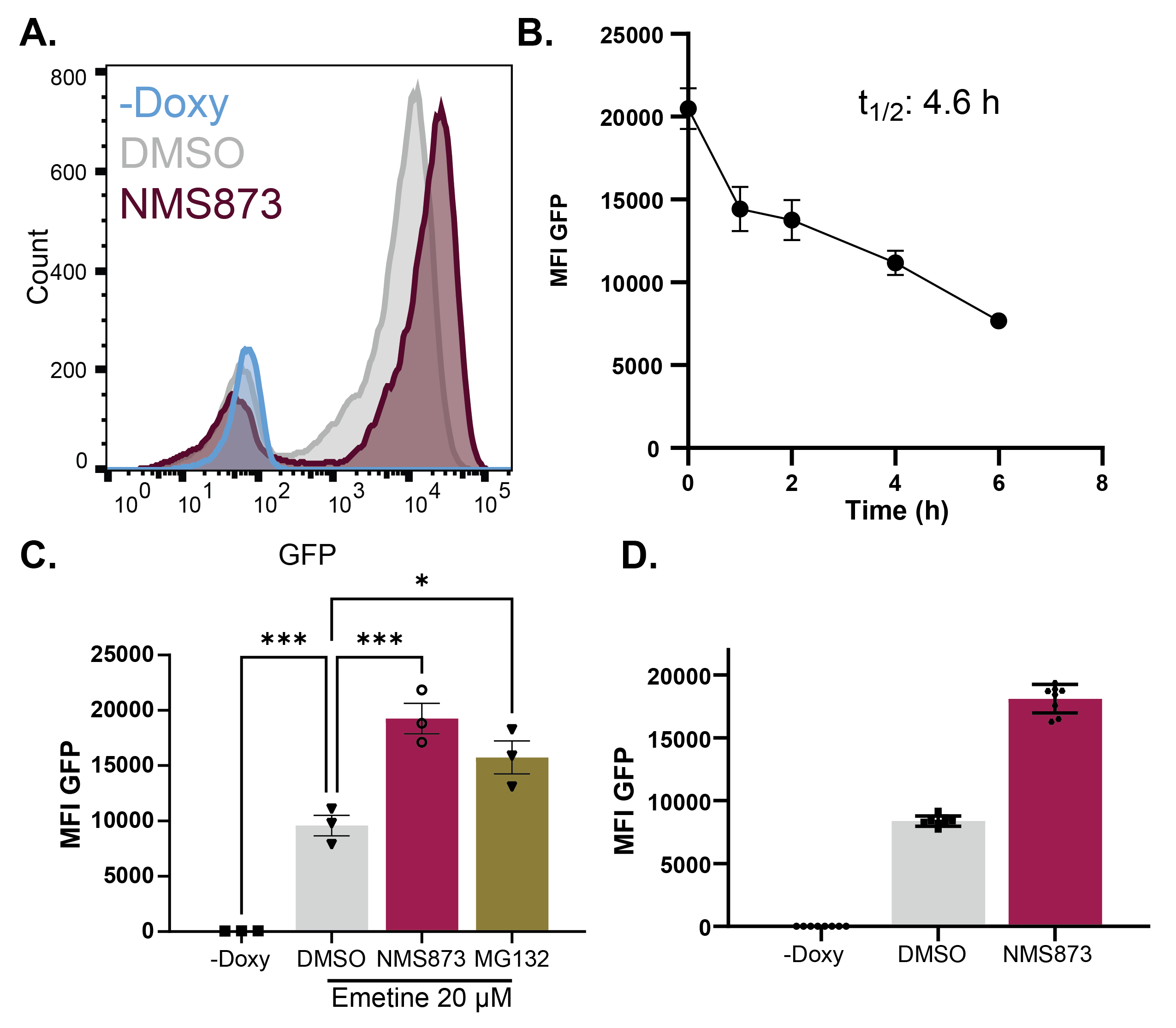

Supplement: Supplementary file 4 — Supplementary Figure 1 [file 41419_2026_8526_MOESM4_ESM.png]

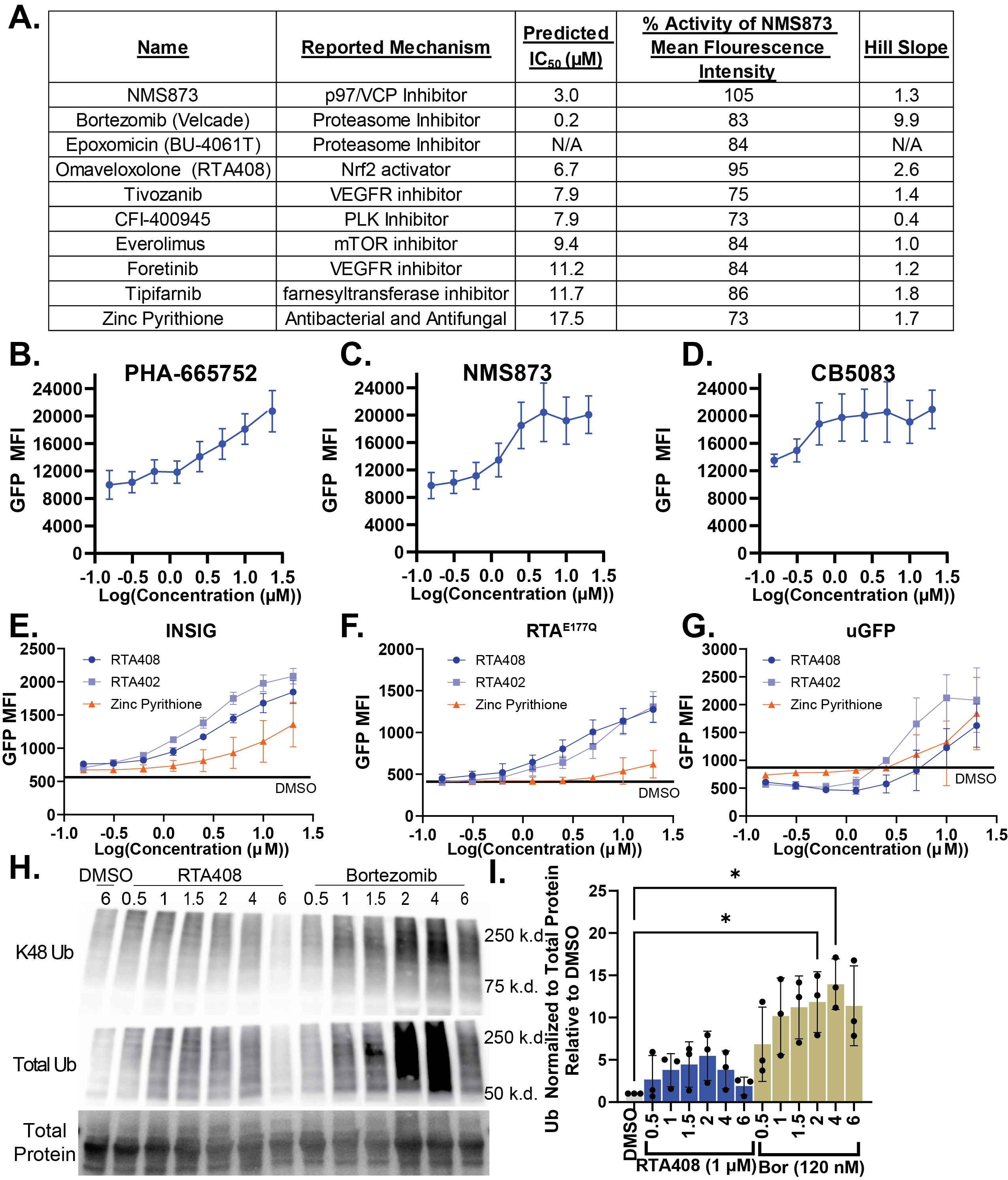

Supplement: Supplementary file 5 — Supplementary Figure 2 [file 41419_2026_8526_MOESM5_ESM.png]

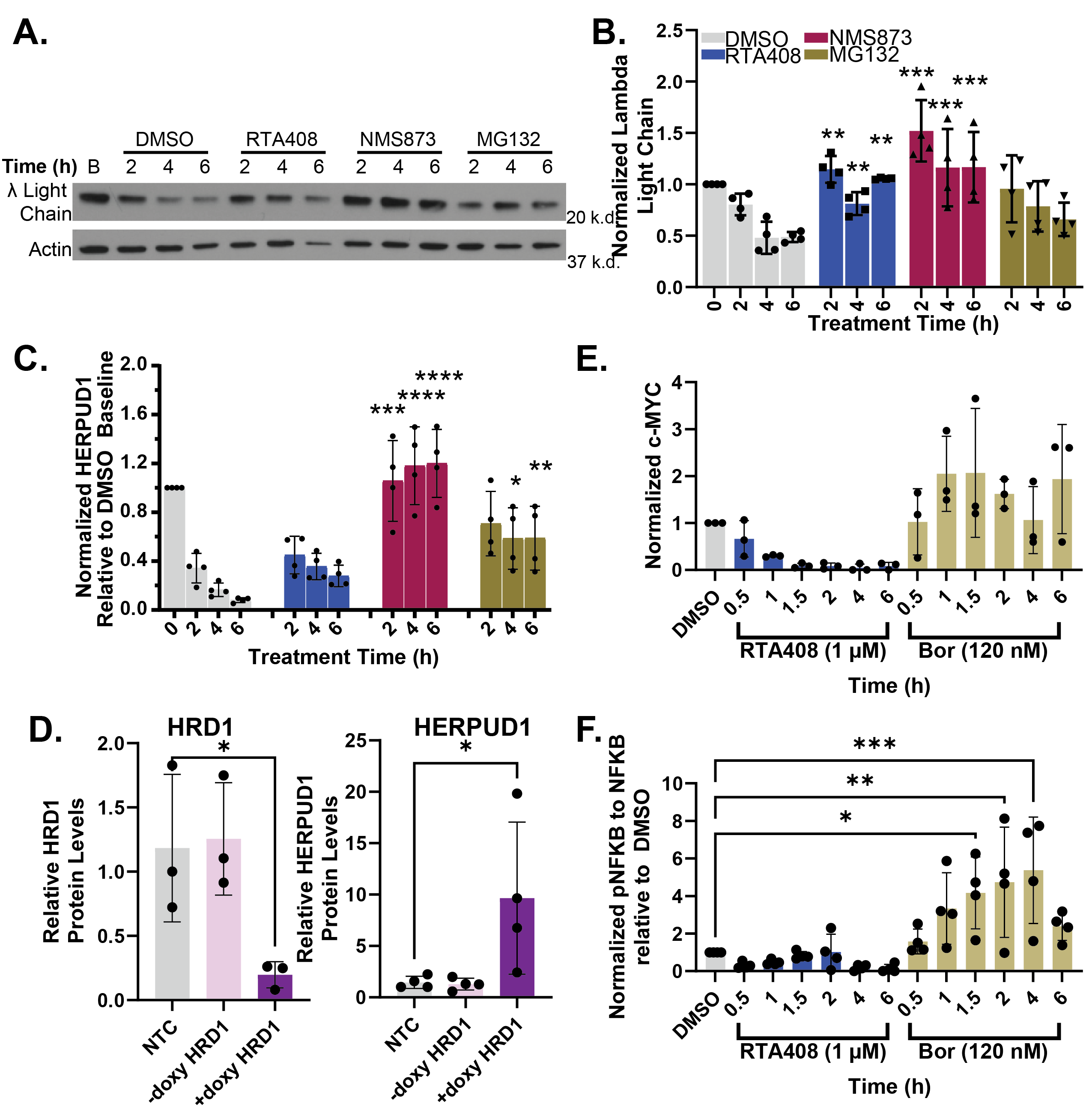

Supplement: Supplementary file 6 — Supplementary Figure 3 [file 41419_2026_8526_MOESM6_ESM.png]

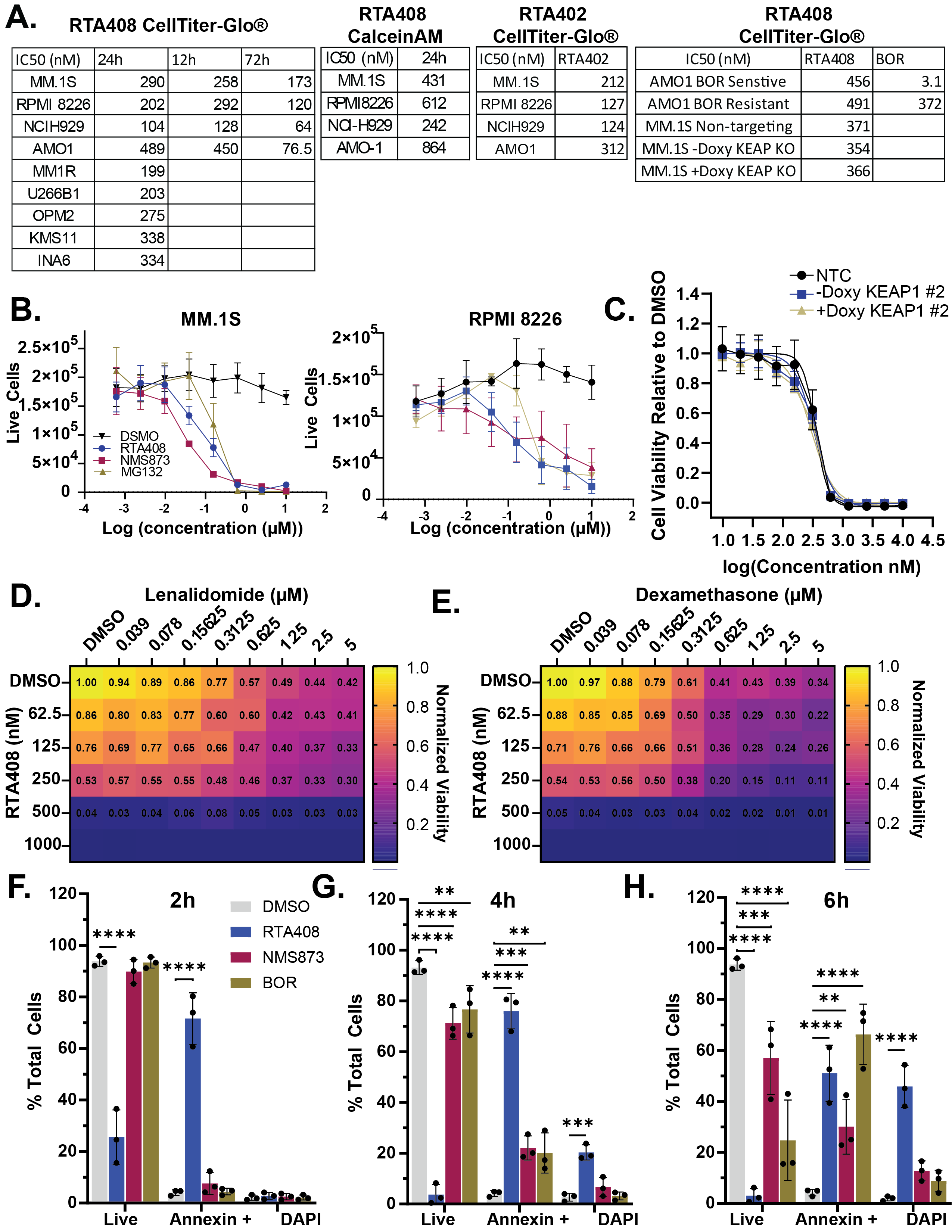

Supplement: Supplementary file 7 — Supplementary Figure 4 [file 41419_2026_8526_MOESM7_ESM.png]

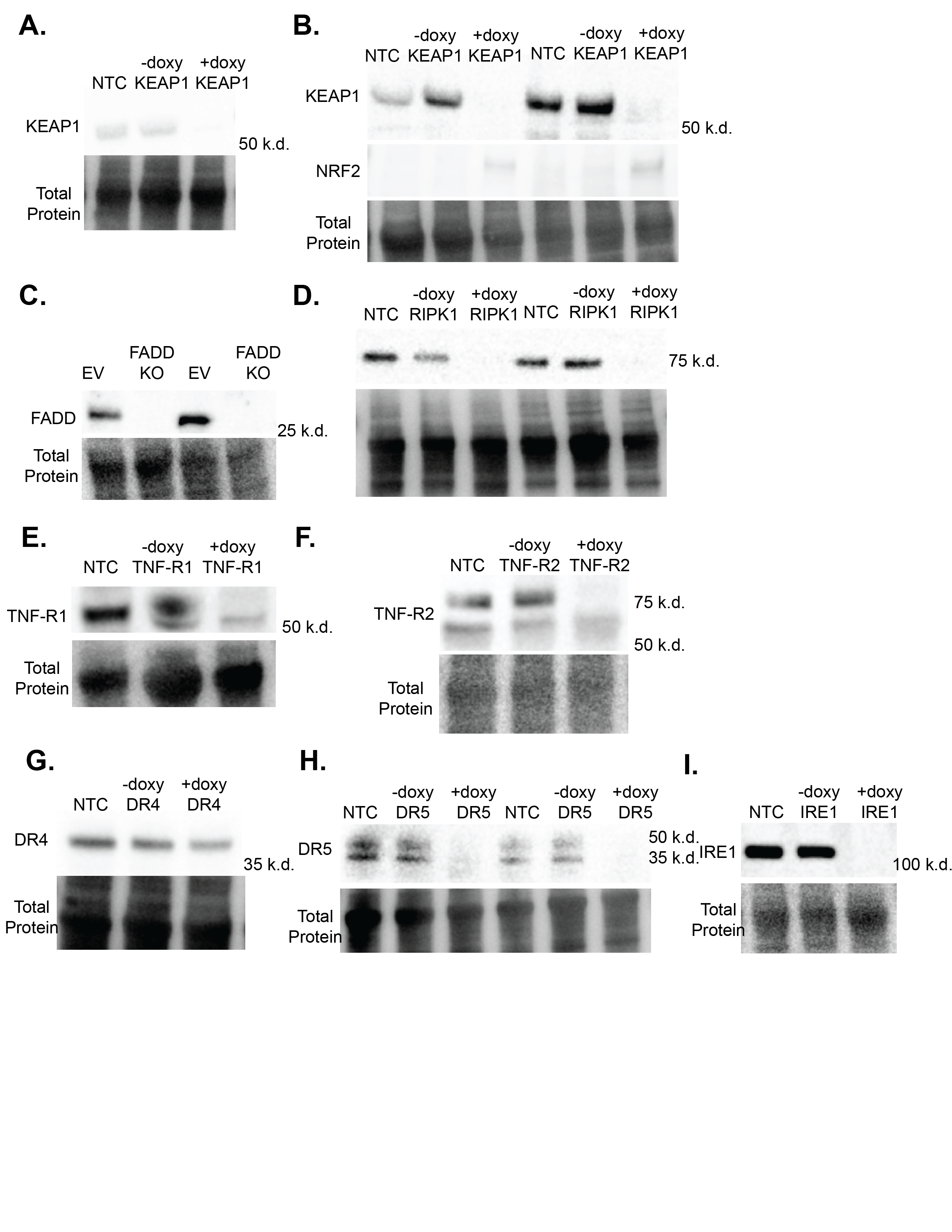

Supplement: Supplementary file 8 — Supplementary Figure 5 [file 41419_2026_8526_MOESM8_ESM.png]

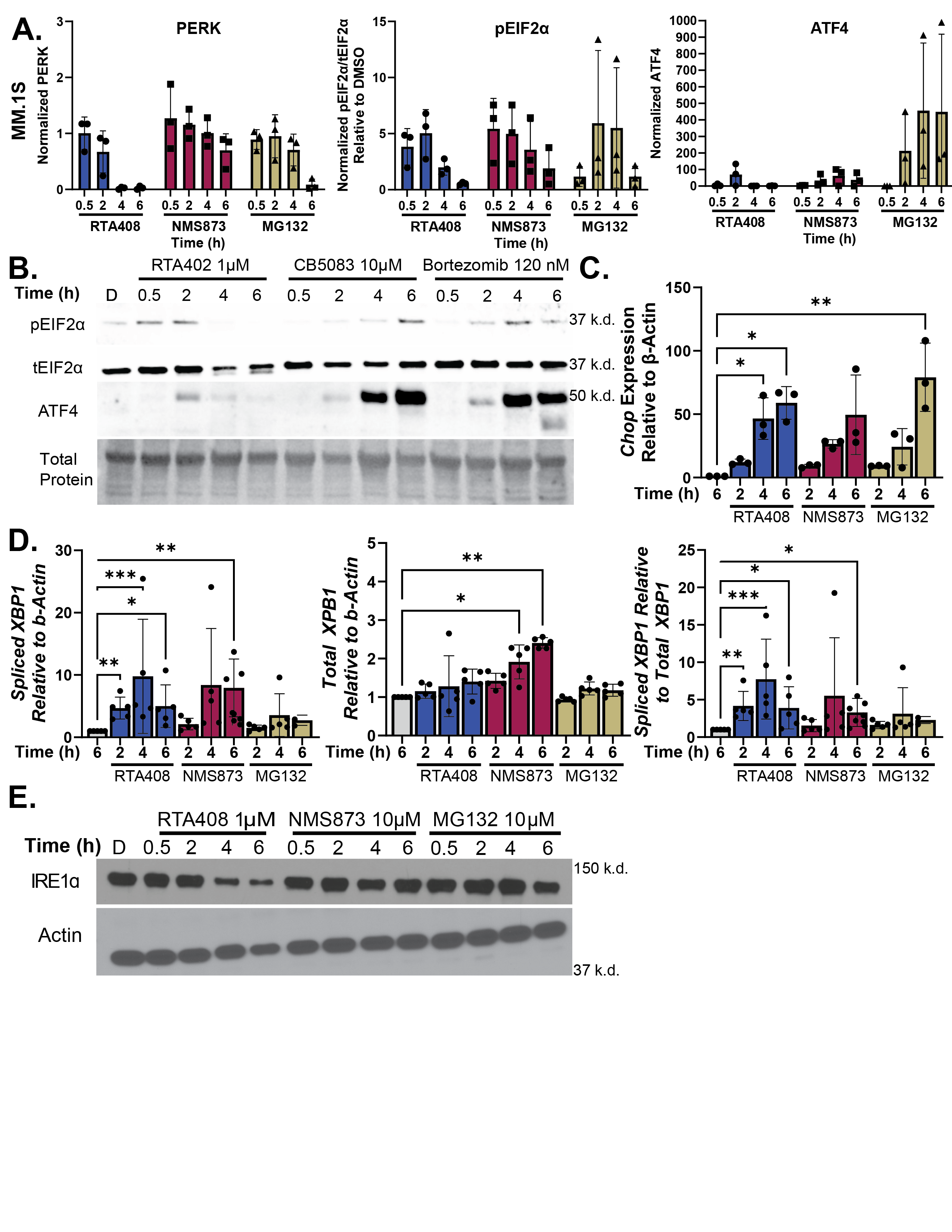

Supplement: Supplementary file 9 — Supplementary Figure 6 [file 41419_2026_8526_MOESM9_ESM.png]

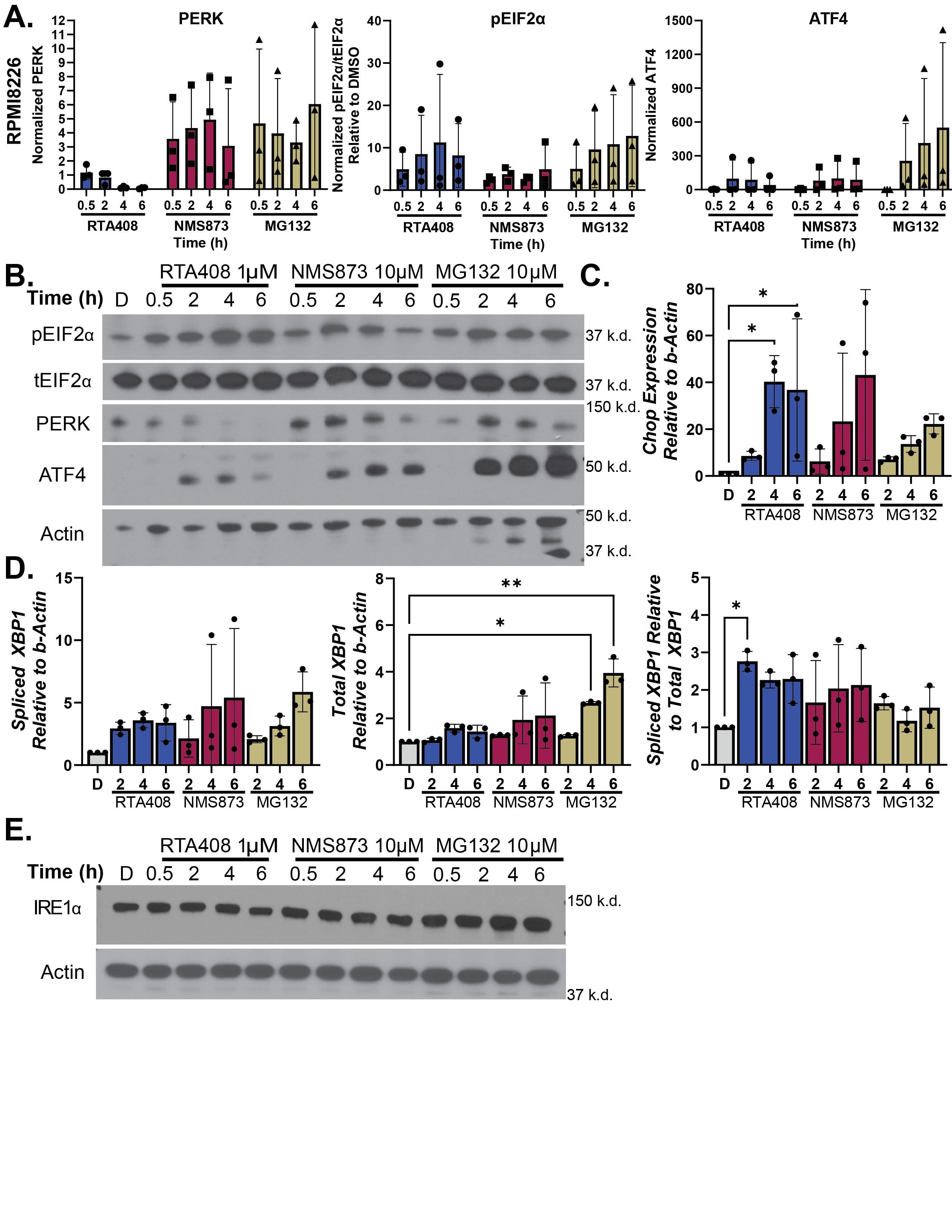

Supplement: Supplementary file 10 — Supplementary Figure 7 [file 41419_2026_8526_MOESM10_ESM.png]

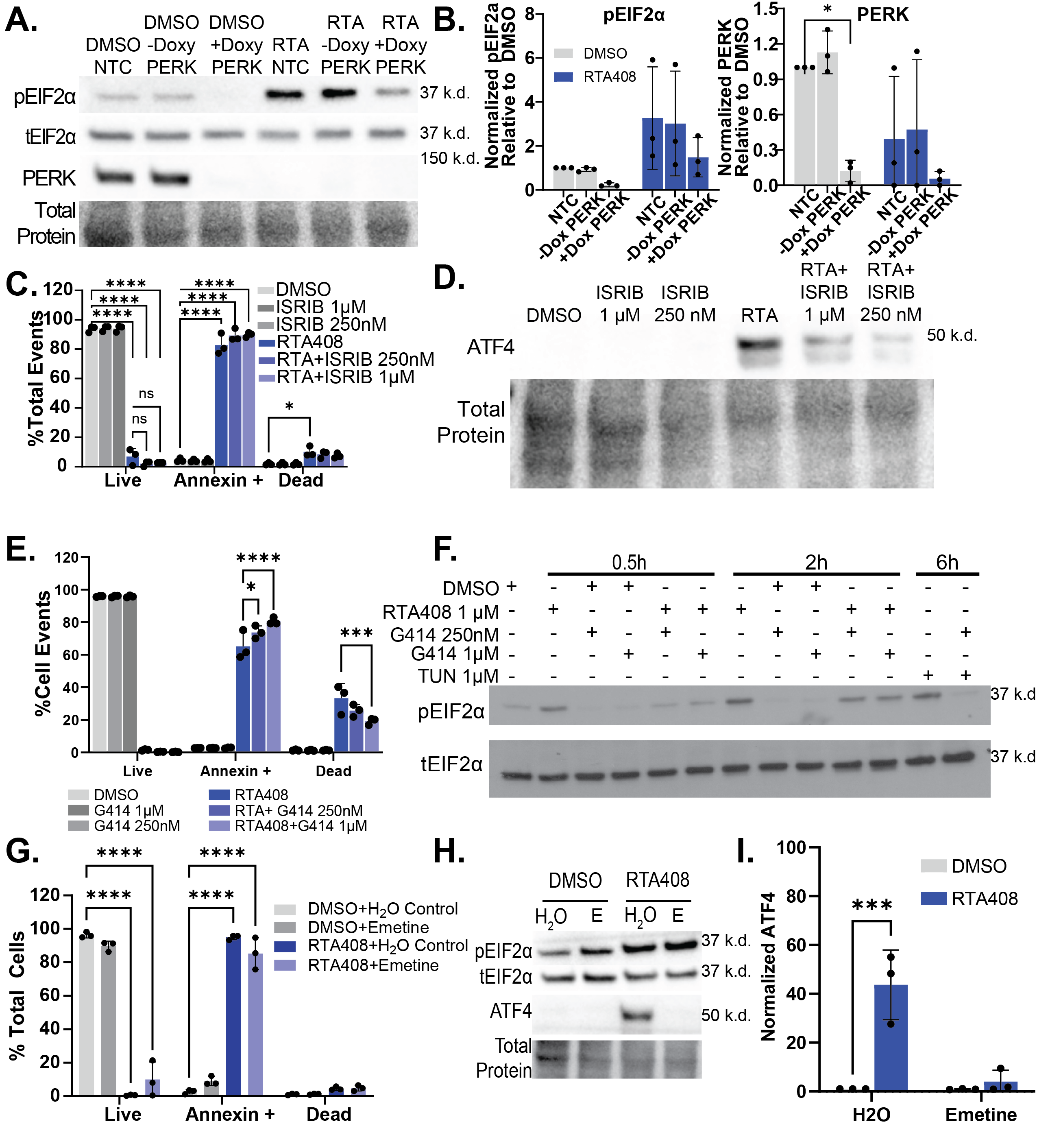

Supplement: Supplementary file 11 — Supplementary Figure 8 [file 41419_2026_8526_MOESM11_ESM.png]

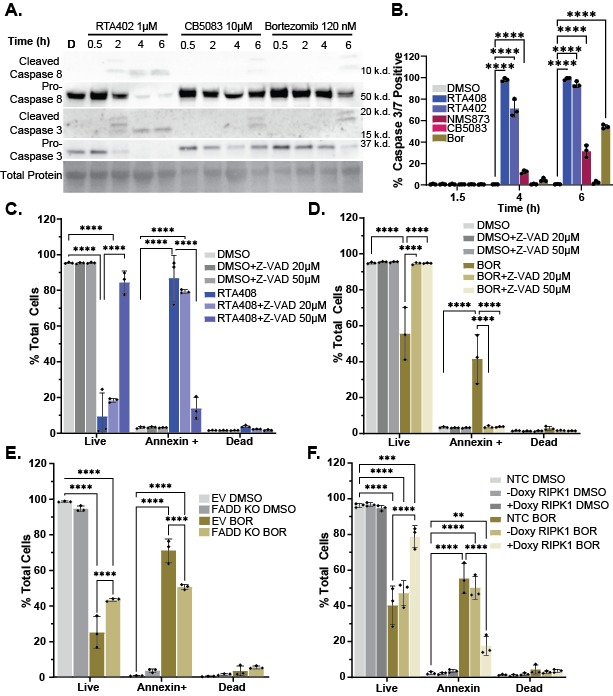

Supplement: Supplementary file 12 — Supplementary Figure 9 [file 41419_2026_8526_MOESM12_ESM.png]

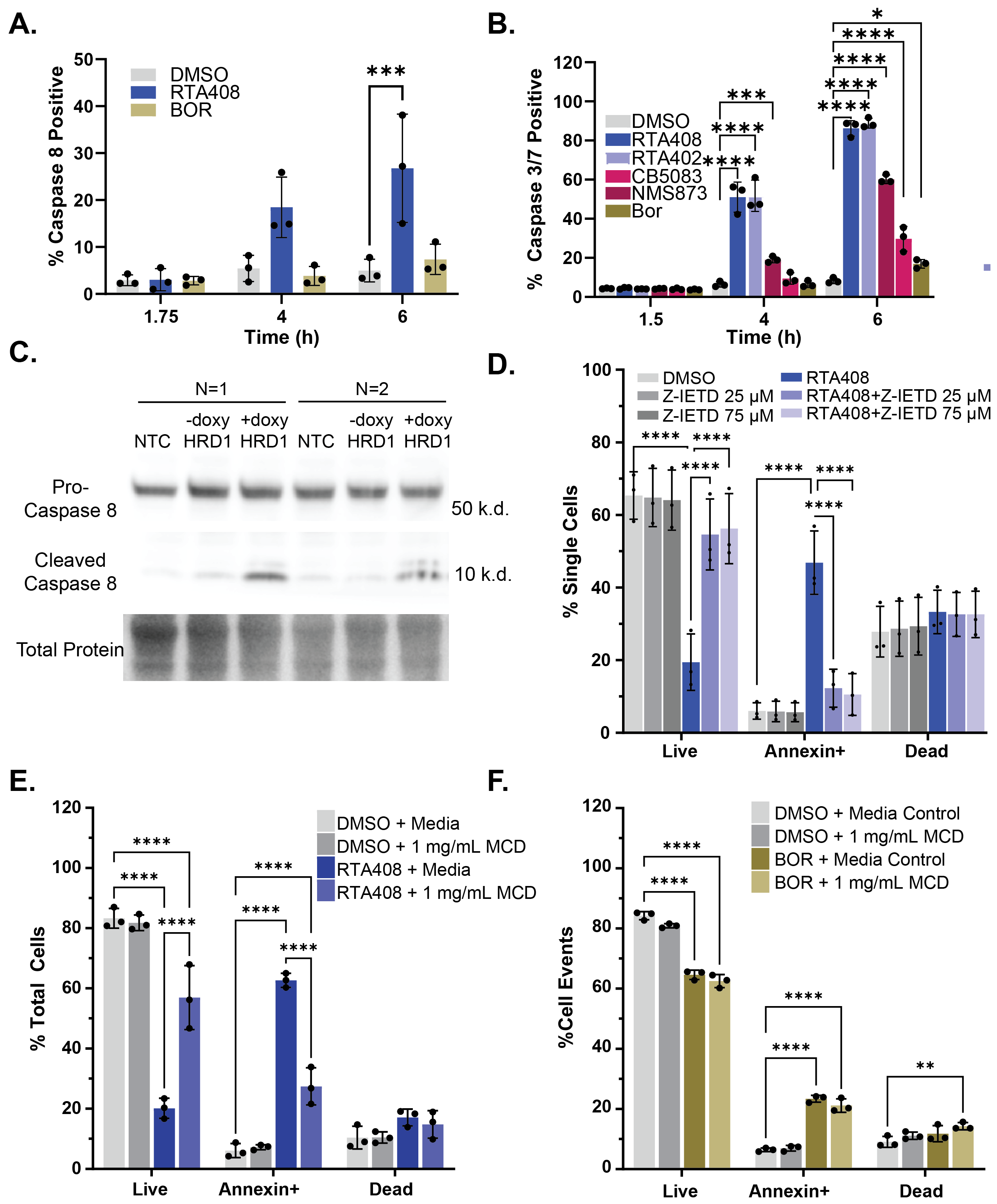

Supplement: Supplementary file 13 — Supplementary Figure 10 [file 41419_2026_8526_MOESM13_ESM.png]

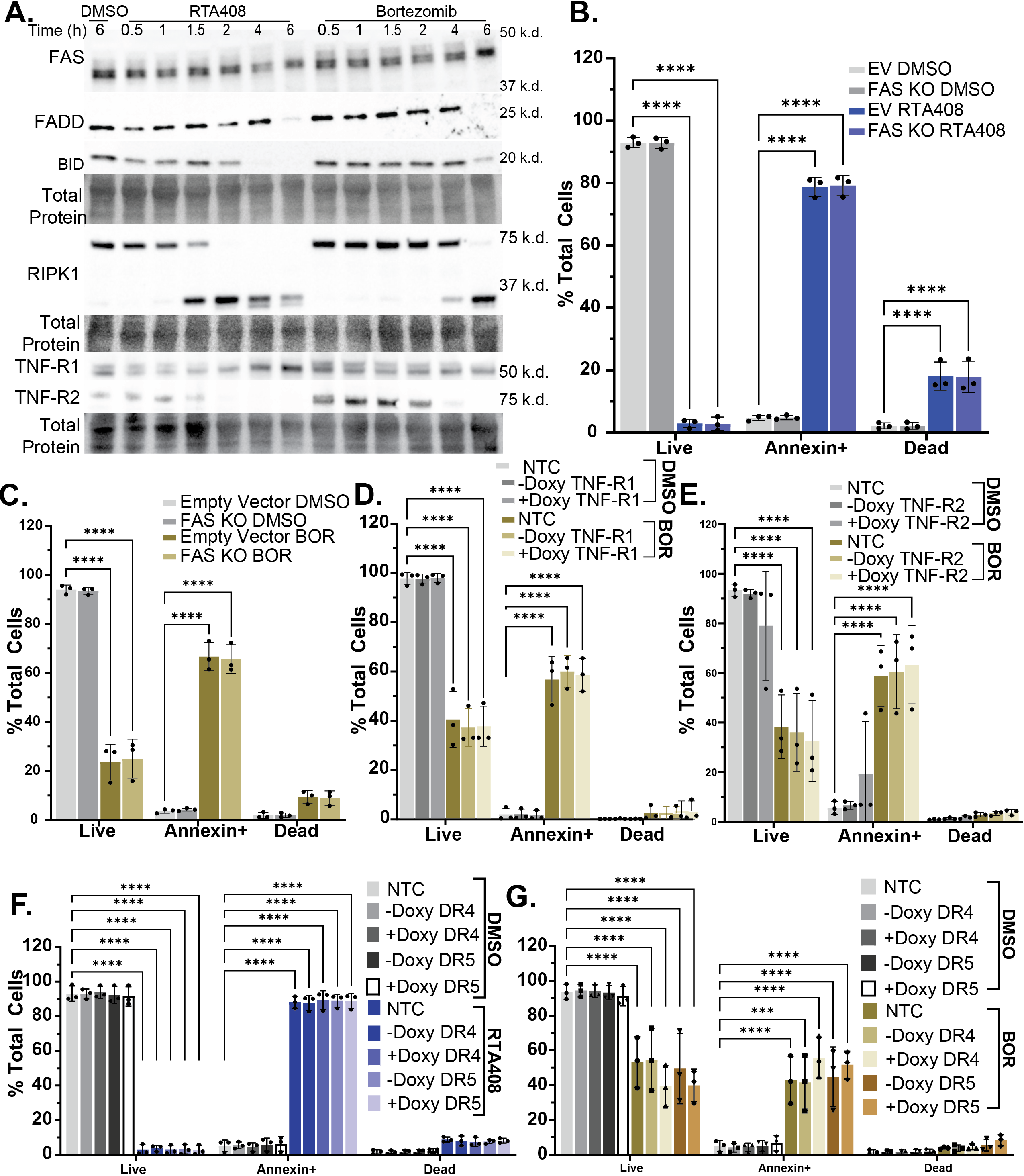

Supplement: Supplementary file 14 — Supplementary Figure 11 [file 41419_2026_8526_MOESM14_ESM.png]

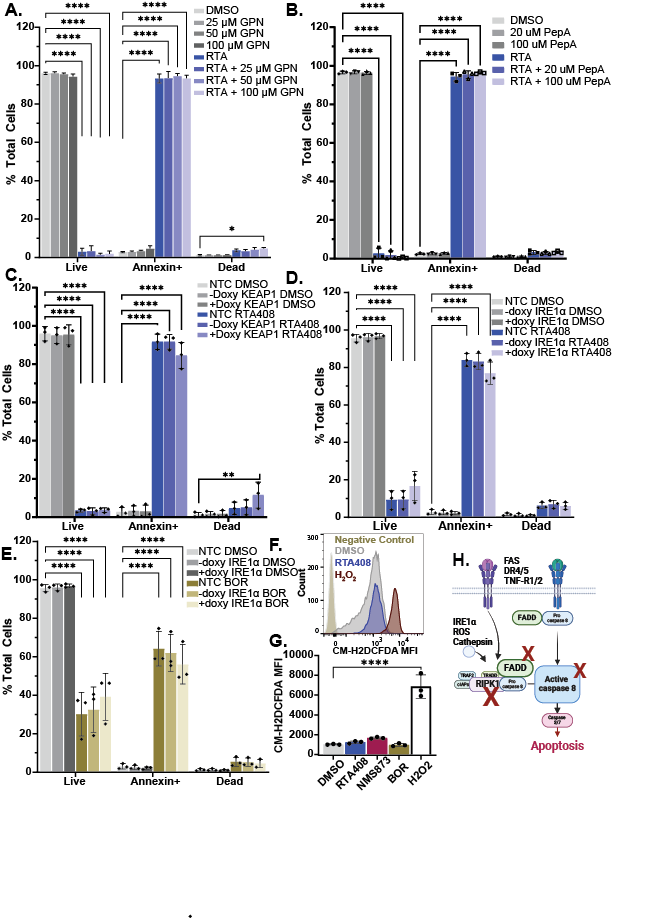

Supplement: Supplementary file 15 — Supplementary Figure 12 [file 41419_2026_8526_MOESM15_ESM.png]

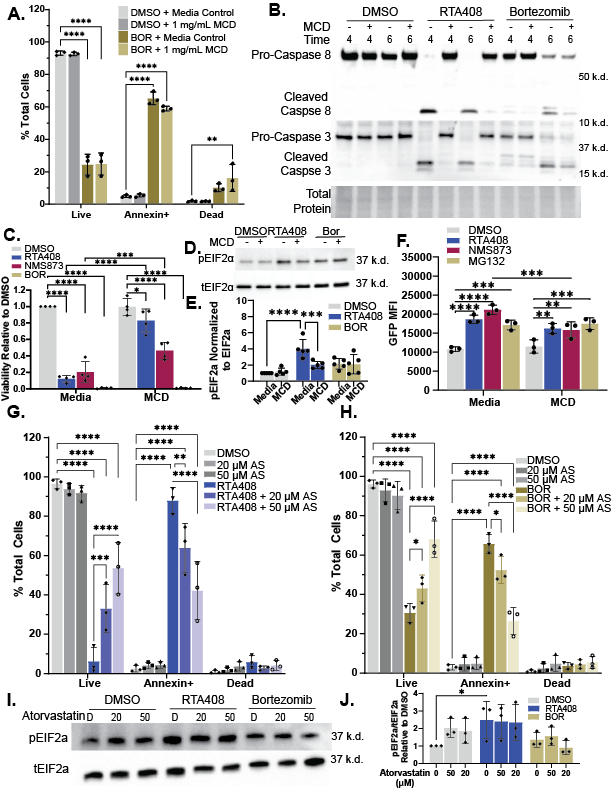

Supplement: Supplementary file 16 — Supplementary Figure 13 [file 41419_2026_8526_MOESM16_ESM.png]

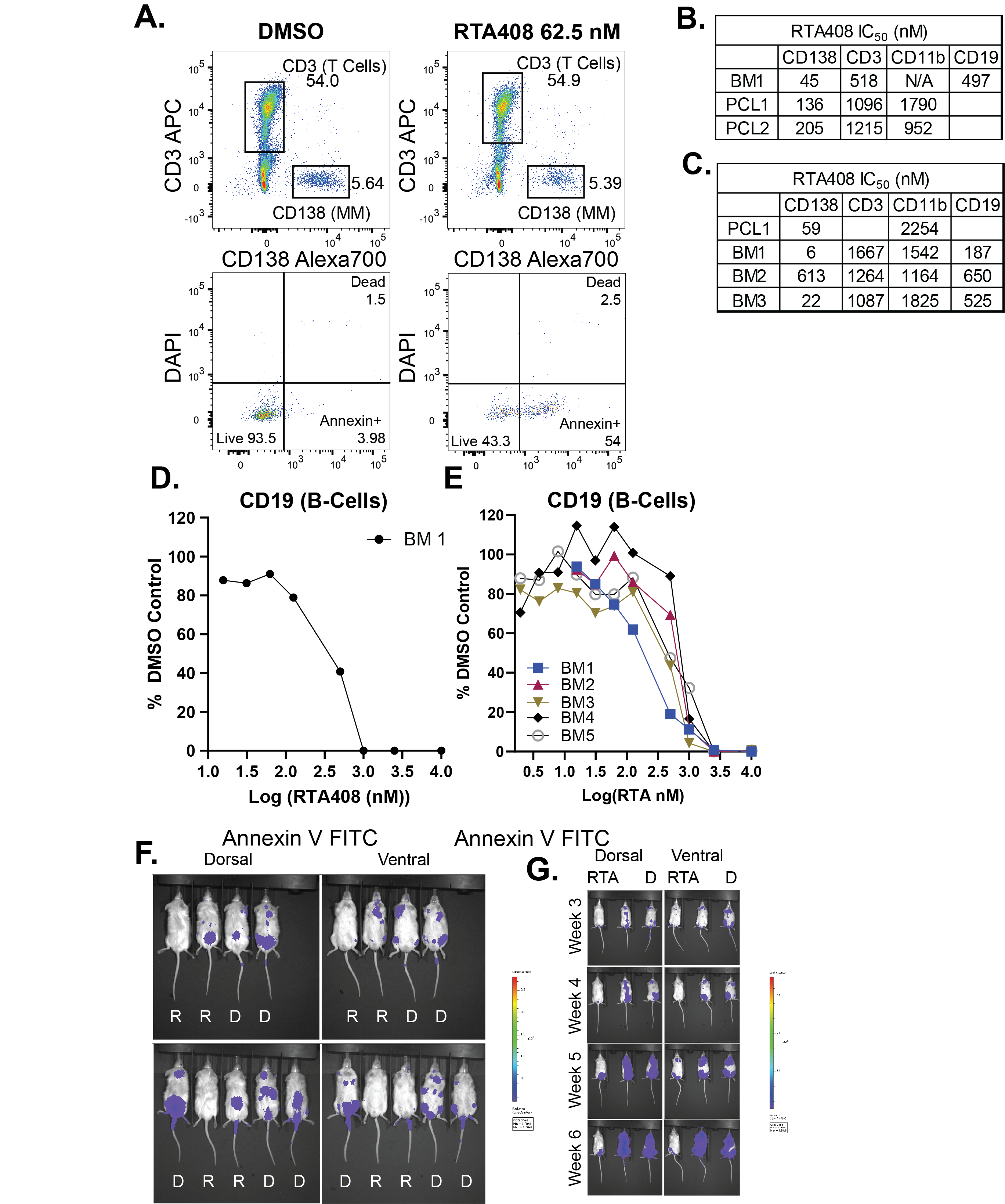

Supplement: Supplementary file 17 — Supplementary Figure 14 [file 41419_2026_8526_MOESM17_ESM.png]

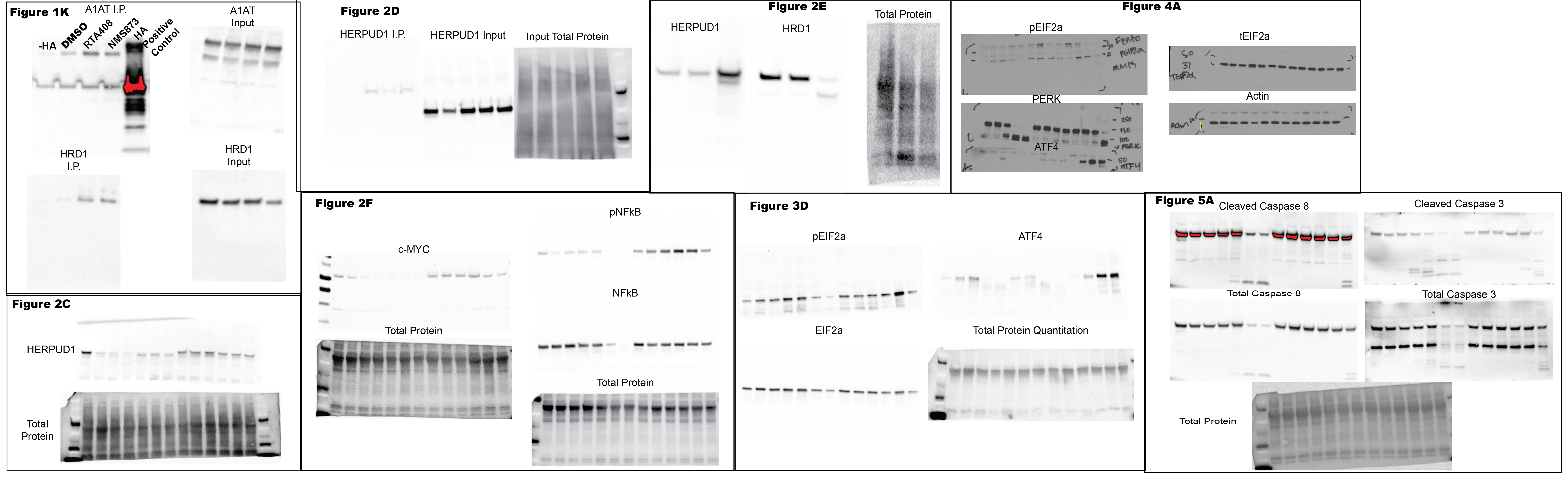

Supplement: Supplementary file 18 — Supp Fig 15 Uncropped Main Western Blot Images [file 41419_2026_8526_MOESM18_ESM.png]

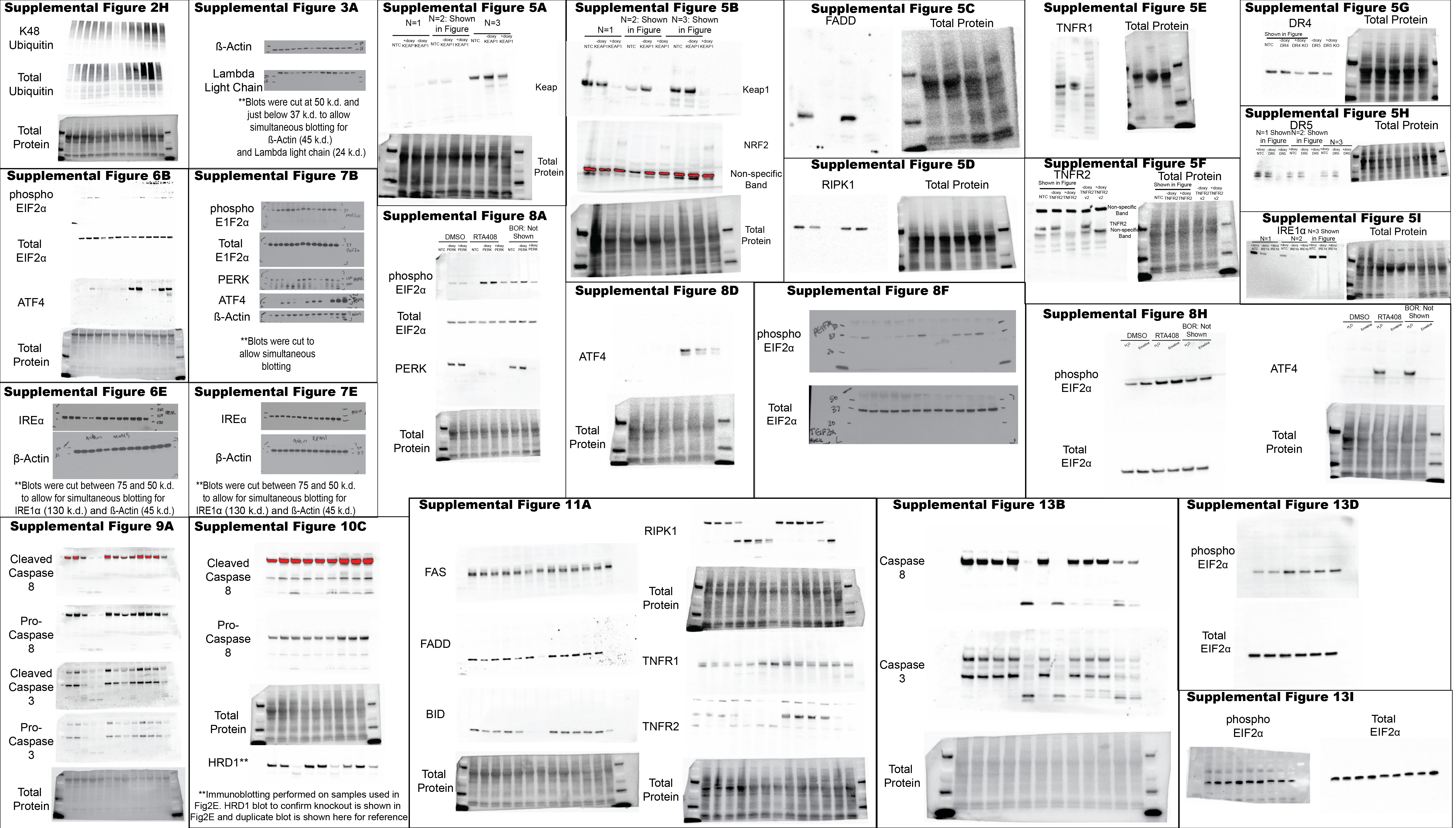

Supplement: Supplementary file 19 — Supp Fig 16 Uncropped Supplementary Western Blot Images [file 41419_2026_8526_MOESM19_ESM.png]
